# Insights into motor control: predict muscle activity from upper limb kinematics with LSTM networks

**DOI:** 10.1038/s41598-025-33696-y

**Published:** 2026-01-05

**Authors:** Marie D. Schmidt, Tobias Glasmachers, Ioannis Iossifidis

**Affiliations:** 1 Institute of Computer Science, Ruhr West University of Applied Sciences, Mülheim an der Ruhr, Germany; 2 Institute for Neural Computation, Ruhr-University Bochum, Bochum, Germany

**Keywords:** Electromyography (EMG), Inertial measurement unit (IMU), Neural networks, Muscle activity, Motion parameters, Swivel angle, Voluntary movement, Artificial generated signal, Generative model, Motor control, Computational biology and bioinformatics, Neuroscience, Motor control

## Abstract

This study explores the relationship between upper limb kinematics and corresponding muscle activity, aiming to understand how predictive models can approximate motor control. We employ a Long Short-Term Memory (LSTM) network trained on kinematic end effector data to estimate muscle activity for eight muscles. The model exhibits strong predictive accuracy for new repetitions of known movements and generalizes to unseen movements, suggesting it captures underlying biomechanical principles rather than merely memorizing patterns. This generalization is particularly valuable for applications in rehabilitation and human-machine interaction, as it reduces the need for exhaustive datasets. To further investigate movement representation and learning, we analyze the impact of motion segmentation, hypothesizing that breaking movements into simpler components may improve model performance. Additionally, we explore the role of the swivel angle in reducing redundancy in arm kinematics. Another key focus is the effect of training data complexity on generalization. Specifically, we assess whether training on a diverse set of movements leads to better performance than specializing in either simple, single-joint movements or complex, multi-joint movements. The study is based on an experimental setup involving 23 distinct upper limb movements performed by five subjects. Our findings provide insights into the interplay between kinematics and muscle activity, contributing to motor control research and advancing neural network-based movement prediction.

## Introduction

Understanding how the brain generates movements and, in particular, planned arm movements is a fundamental question in motor control research. Motor control encompasses a broad spectrum of mechanisms, from neural activity in different areas and levels to the representation of movement parameters. Motor control is orchestrated by a distributed network of interconnected regions, but is not limited to, the primary and premotor cortices, supplementary motor area, cerebellum, basal ganglia, and spinal cord. These regions work together to coordinate the planning, initiation, and execution of movements^1^. Behavior and computational studies have provided information on how the central nervous system plans and executes movements with efficient control strategies.

One approach to modeling motor cortical activity is through dynamical systems and recurrent neural networks (RNNs), which suggest that preparatory activity in the motor cortex establishes initial conditions that evolve predictably to generate reaching movements^2–5^. Pioneering studies by Georgopoulos et al. demonstrated correlations between hand movement parameters, such as direction, speed, and acceleration, with neural activity in the motor cortex during reaching tasks^6–9^. These high-level kinematic representations contrast with lower-level control strategies that directly influence muscle activity and force generation^10–12^.

A key challenge in motor control is managing redundancy in the human motor system. There are many possible ways to achieve the same movement outcome. Latash introduced the principle of abundance, suggesting that motor variability is not noise but a functional strategy that allows adaptability^13^. Bernstein’s theory of muscle synergies proposes that the nervous system simplifies control by activating groups of muscles as functional units rather than individually controlling each muscle^14,15^. These concepts align with hypotheses of modular motor organization, which describe movement generation through coordinated structures such as motor primitives, modules, and synergies^16^. Tessari et al. recently proposed the synergy expansion hypothesis, offering a new perspective on how motor synergies develop and might be functionally employed. The hypothesis is structured around three propositions. Mechanical Proposition: Humans can possess more synergies than controlled features, creating an overcomplete set of motor solutions that enhances adaptability. Developmental Proposition: Motor skills evolve from crude, primordial synergies toward highly specialized synergies through learning and refinement. Behavioral Proposition: Learned synergies are stored and recruited as needed, allowing flexible execution of motor tasks^17^. The level of this coordination remains a topic of debate, with evidence pointing to both cortical and spinal contributions^18,19^. Although the exact mechanism remains elusive, it is widely accepted that these organizational structures, coordinated by motor cortical areas, enable motor control^20^.

Dynamic systems and recurrent neural networks have been widely used to model various aspects of motor control. In dynamic system models, the evolution of a system depends on its initial conditions, which, in the context of motor-related brain areas, include somatosensory inputs describing limb position, spatial orientation relative to the body and environment, potential target locations, and cognitive expectations regarding object properties such as weight and texture. These initial conditions are further refined by motor planning processes and prior experience. In this study, we approximate these inputs using kinematic data in the form of high-level movement representations. These serve as inputs to our recurrent model, providing a fundamental approximation of the underlying dynamic processes. Our model estimates the corresponding muscle activity, effectively bridging the gap between movement planning and execution. Prior research has demonstrated the feasibility of predicting muscle activity from kinematic data, revealing the inherent link between these variables^21–26^.

In this framework, our model serves as an abstract representation of motor-related neural processes. We employ a Long Short-Term Memory (LSTM) network (Section LSTM model), a type of RNN particularly well-suited for capturing temporal dependencies within sequential data. LSTMs have been shown to effectively model kinematic data and muscle activity^26^. Furthermore, previous research suggests that end effector (EEF) data provide a more efficient representation of movement^27^ compared to individual joint angles, enabling LSTMs to better capture the temporal dynamics of movement^26^. Previous work provides the foundation for the subsequent work, we have addressed questions such as which model architecture is most suitable, which kinematic input type is preferable, how much data is required, and whether models should be trained individually or across subjects. This study explores the potential of LSTM-based models trained on EEF data to predict muscle activity, with a particular focus on their ability to generalize beyond known movements to novel, previously unseen movements. The model’s ability to infer muscle activation for untrained movements suggests that it does not merely memorize movement patterns but learns underlying biomechanical principles, making it a generative approach applicable to real-world motor tasks.

In addition, we explore the impact of incorporating elbow information to reduce redundancy in arm kinematics (Section Redundancy in arm kinematics: The impact of the swivel angle). Although the end effector data alone can represent multiple possible arm configurations, it does not uniquely describe a single posture; it can lead to varying neuromuscular activation patterns. To address this, we introduce the swivel angle as an additional kinematic input, representing the rotational degree of freedom of the elbow about the shoulder–wrist axis. Formally, it is defined^28,29^ as the angle between the plane spanned by the shoulder, elbow, and wrist joints, and a reference plane defined by the shoulder and wrist (Fig. 9). By including this information, we aim to reduce redundant arm configurations and examine how this affects the model’s ability to predict muscle activity across varying arm postures.

Further, we want to investigate the influence of movement type. We examine whether training on simpler, near one-dimensional movements, or more complex, multi-joint movements leads to better generalization in modeling (Section Movement complexity). This distinction arises from a fundamental question: How to control multiple degrees of freedom. Is it more effective to first learn the degrees of freedom for each individual joint and subsequently combine them to generate all possible multi-joint movements, or is it more efficient to learn multi-joint coordination from the start? These two perspectives originate from different theoretical frameworks. The first aligns with a modular viewpoint, closely related to motor primitive theory, which posits that complex movements are composed of a limited number of primitives, which do not need to be universal but can be task specific^30,31^. The primitives can be of kinematic and or dynamic nature depending on the extraction criteria. In contrast to the muscle synergy, movement primitives are based on a behavioral level^32^. This concept is also connected to submovements, which are defined as elementary kinematic units, characterized by a single cycle of acceleration and deceleration. This is typically represented by bell-shaped velocity profiles in the trajectory of the end effector. Krebs et al. provided evidence for this idea by demonstrating that, after a brain injury, continuous reaching trajectories can be broken down into distinct, stereotyped submovement elements. This suggests that such primitives may serve as a fundamental organizational principle in human motor control^33^. We explore two distinct input structures for our network models. One involves leaving movements intact, while the other entails segmenting each movement into sequences. For the segmentation, we adopted a related strategy; instead of relying on bell-shaped velocity profiles, we employed bell-shaped acceleration profiles based on the inflection points of the end effector velocity profile. This approach allows movements to be decomposed more finely, including simple movements, thereby yielding a greater number of kinematically homogeneous sequences. Furthermore, this approach is physiologically motivated, as acceleration is more directly linked to the underlying muscle forces. This approach aligns with the theory supporting the notion that complex movements can be deconstructed into simpler components. In contrast, early motor development in biological systems suggests a different strategy. Infants typically display multi-joint reaching behaviors^34^ and exhibit first gross motor skills as a foundation for fine motor skills^35^, implying that neuromuscular coordination is shaped by global movement patterns rather than isolated joint control. This view aligns with the synergy expansion hypothesis, which suggests that humans may begin with a preliminary set of coarse motor synergies that are gradually refined and expanded into more specialized and advanced motor skills over the course of development^17^. Furthermore, the muscle activation patterns of a single-joint movement may differ when the same joint is involved in a coordinated multi-joint task, indicating that context-dependent neuromuscular control plays a crucial role in movement execution. These considerations emphasize the importance of understanding whether a model trained on simple, single-joint movements can generalize effectively to complex movements or whether learning multi-joint coordination from the outset yields better predictive performance.

We designed a comprehensive study that captured both kinematic and muscle activity data from 23 distinct upper limb movements (Table 1) performed by five subjects. These movements ranged from simple, single-joint movements, such as shoulder abduction, to complex, multidimensional daily activities, like reading a watch. The recorded kinematic data included end effector position and orientation, while muscle activity was measured using surface electromyography (sEMG) from eight upper limb muscles. Consistent with prior work, we use the term “muscle activity” to refer to the neuronal signal at the muscle membrane, measurable through surface electromyography (EMG)^25–27,36,37^.

The following provides a brief overview of existing approaches for predicting muscle activity from movement data, covering both biomechanical models and machine learning-based methods. Analytical methods rely on biomechanical models to compute muscle activity from movement trajectories^21,38^, while machine learning approaches often involve neural networks learning the relationship between motion and muscle activity directly from data. Most models use joint angles (typically shoulder and elbow) or hand EEF trajectories as input^21–25^. Studies vary widely in task complexity, ranging from simple to some 3D movements, and typically record from 8–12 muscles across 5–9 subjects. The most common machine learning approach involves feedforward neural networks^24,25,27^. Johnsen and Fuglevand compared various probabilistic approaches and found that a dynamic neural network, essentially a feedforward neural network with time-delayed inputs, performed well, achieving an average accuracy of r^2^= 0.40 for random 3D movements^36^. Other studies also report promising results using feedforward neural networks: Rittenhouse et al. reached r^2^= 0.66 for a press-up task^24^, while Tibold and Fuglevand reported R^2^=0.43 for both loaded and unloaded random 3D movements^27^. Pohlmeyer et al. investigated the prediction of EMG activity across four muscles from motor cortex signals during a button press task. Using a linear model, they achieved test set accuracies with R^2^ values ranging between 0.60 to 0.70^39^. Similarly, Nicolelis et al. employed ensemble activity from the dorsal premotor, primary somatosensory, and posterior parietal cortices to predict EMG signals for four muscles using a linear model, achieving an r of 0.52^40^. The relatively low R^2^ values indicate that a portion of the variance remains unexplained. However, this level of performance is consistent with previous studies predicting EMG from kinematic data using data-driven models. It likely reflects the inherent complexity and variability of muscle activation, which depends not only on kinematics but also on unobserved factors.

One might question why the R^2^ values remain in the range of $0.4-0.7$ for linear and non-linear models, which can capture complex temporal dependencies; their predictive performance is fundamentally limited by the information contained in the input features. EMG activity is determined not only by kinematics but also by additional factors such as muscle forces and neural control.

Understanding the relationship between upper-limb kinematics and muscle activity can provide functional insights into how movement and muscle activation are coupled, even if the underlying neural control processes remain inaccessible to the model. Such insights are not only crucial for advancing our understanding of motor control, but they also have significant implications for real-world applications in rehabilitation, prosthetics, and human-machine interaction. This understanding offers a promising approach to predicting muscle activity from movement data, which can reduce the need for exhaustive datasets and ensure reliable performance, even with limited training data. However, it should be noted that these results are based on healthy subjects, and transferring them to patients with neurological disorders would present additional challenges and require further research. Additionally, the model’s ability to generalize to new, unseen movements enhances its potential for real-world applications, where individuals frequently perform variations of known movements.

## Methods

In this section, we outline the fundamental aspects of our study, including its design, technical details, and procedure. We then introduce our LSTM model itself, the training strategies employed, and the evaluation metrics used to assess its performance.

### Study design

The study involves five healthy participants (2 female, 3 male, aged 26 ± 2 years), all provided informed written consent to participate in the study, approved by the Ethics Committee of Ruhr-University Bochum, and the experiment adhered to ethical guidelines. All subjects completed 23 tasks, each comprising 18 repetitions of unloaded, dynamic upper-limb movements (sometimes referred to as isotonic). Isotonic movements are characterized by muscular contractions that lead to changes in muscle length, thereby producing movement at the corresponding joints. The movements analyzed in this study are categorized into two groups: simple and complex movements, see Table 1. The Table also lists all movements and indicates whether each subject performed them. Some movements are missing, primarily due to electrode loosening or incorrectly performed tasks, such as uncontrolled return movements where participants did not actively guide the arm back to the resting position.

**Fig. 1:**
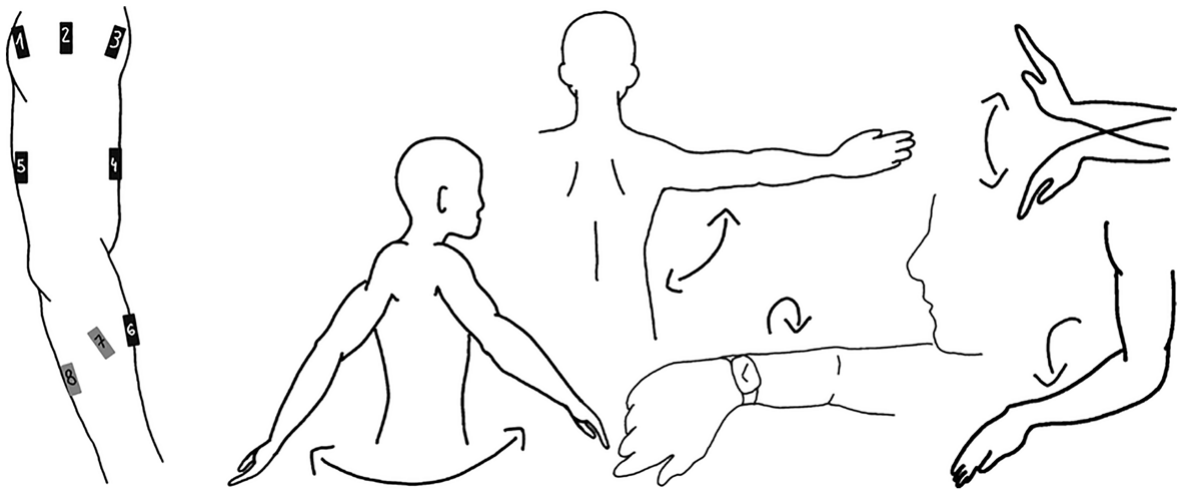
Electrode placement deltoid posterior (1), medial (2), anterior (3), biceps short head (4), triceps brachii lateral head (5), pronator teres (7), flexor carpi ulnaris (8), and extensor carpi ulnaris (6). Illustrates some of the tasks arm movements, including shoulder flexion and extension, shoulder abduction, reading the clock, wrist extension and flexion, and wrist pronation, modified from^42,43^.

Simple movements are defined as movements primarily occurring within one joint in a single plane, making them approximately one-dimensional. Participants were not rigidly constrained but were guided to perform the movements within the intended plane. Simple movements (Fig. 1) include shoulder flexion-extension, where the arm is raised in the sagittal plane to an angle of $90^\circ$ and lowered to $-30^\circ$, and shoulder abduction, involving the lifting of the arm in the frontal plane to $90^\circ$ before returning to a resting position at $0^\circ$. Elbow flexion-extension involves bending and straightening the elbow, and moving the forearm towards and away from the upper arm. Wrist movements include flexion-extension, in which the hand tilts up and down relative to the forearm, and pronation, where the forearm is rotated to position the palm downward.

In contrast, many complex movements have several degrees of freedom through the involvement of two or more joints. Complex movements are multi-dimensional and do more closely resemble natural, everyday actions. Examples of complex movements include the arm-rolling movement, where the forearms rotate over one another in front of the body. A breaststroke movement simulates the arm movement of the breaststroke swimming technique, and a relay handover transfers an imaginary object, involving shoulder extension and wrist pronation, behind the back. Additionally, complex movements include reading a watch (Fig. 1), where the forearm is lifted in front of the face, a diagonal reach, where the right arm crosses the body to tap the left arm at different heights, a waving gesture, and drawing a circle or a line in the air, always starting from a position in front of the body.

**Table 1:** All tasks listed, including simple and complex movements are performed to the natural joint maximum. Movements labelled (mix) involve arbitrary endpoint changes, while those labelled ($90^\circ$) involve stopping at that degree. The $\times$ indicates whether the movement is available in the dataset of each subject.

	movements	1	2	3	4	5
simple movement	shoulder flexion ($90^\circ$)	$\times$	$\times$	$\times$	$\times$	$\times$
	shoulder flexion (mix)	$\times$	$\times$	$\times$	$\times$	$\times$
	shoulder extension	$\times$	$\times$	$\times$	$\times$	$\times$
	shoulder abduction ($90^\circ$)	$\times$	$\times$	$\times$	$\times$	$\times$
	shoulder abduction (mix)	$\times$	$\times$	$\times$	$\times$	$\times$
	elbow flexion	$\times$	$\times$	$\times$	$\times$	$\times$
	elbow flexion (mix)	$\times$	$\times$	$\times$	$\times$	$\times$
	elbow flexion with a supinated forearm	$\times$	$\times$	$\times$	$\times$	
	wrist flexion	$\times$	$\times$	$\times$		$\times$
	wrist extension	$\times$	$\times$	$\times$	$\times$	$\times$
	wrist pronation	$\times$	$\times$	$\times$	$\times$	$\times$
	shoulder abduction & flexion		$\times$	$\times$	$\times$	$\times$
complex movement	shoulder abduction with simultaneous elbow flexion	$\times$	$\times$	$\times$	$\times$	$\times$
	shoulder flexion with simultaneous elbow flexion	$\times$	$\times$	$\times$		$\times$
	shoulder abduction with simultaneous wrist extension	$\times$	$\times$	$\times$	$\times$	$\times$
	arm roll		$\times$	$\times$	$\times$	$\times$
	breaststroke	$\times$	$\times$	$\times$	$\times$	$\times$
	relay handover	$\times$	$\times$	$\times$	$\times$	$\times$
	reading a watch	$\times$	$\times$	$\times$	$\times$	$\times$
	diagonal reach	$\times$	$\times$	$\times$		$\times$
	waving gestures	$\times$	$\times$	$\times$	$\times$	$\times$
	drawing a circle		$\times$	$\times$	$\times$	$\times$
	drawing a line		$\times$	$\times$	$\times$	$\times$

All movements initiate and conclude in a resting position, in which the arm rests parallel to the side of the body, with participants instructed to execute controlled movements without relying on gravity for the return phase. Participants were guided by a visual interface on a screen, initiated with a 4 s ’resting window’ and followed by a repeating 7.5 s instruction window for every motion. A 60 s resting period ensued after each task to mitigate muscle fatigue. The speed of the motions was not directly imposed. Instead, participants were instructed to select their own comfortable pace, as long as the execution remained within the predefined time window and maintained one smooth, controlled movement.

The muscle activity is recorded at a rate of 2222 Hz using the Trigno Wireless EMG System (Delsys Inc., Boston, MA, USA) with eight electrodes placed on the upper right arm. The preparation and positioning of electrodes followed the guidelines outlined in the SENIAM manuscript^41^. The selection of muscles included in our recordings captures the primary movers of the upper limb, directly involved in the executed reaching tasks, and provides representative coverage of shoulder, elbow, and wrist joint actuation. The recorded muscles include deltoid anterior, medial, and posterior, biceps short head, triceps brachii lateral head, pronator teres, flexor carpi ulnaris, and extensor carpi ulnaris. The kinematic data are captured using the IMU-based Xsens Motion Capture (Xsens Technologies B.V., P.O. Box 559, 7500 AN Enschede, Netherlands) in an upper body configuration featuring 11 sensors with a sampling rate of 60 Hz. The EMG system is start and stop synchronized with the motion tracking from Xsens Motion Capture via the Delsys trigger box. To align both data streams to a common sampling rate, the EMG signal is processed with a root mean square, sampled at 60 Hz to match the Xsens Motion Capture timestamps. Specifically, a 200 ms window is centered around each Xsens timestamp. Subsequently, all EMG timestamps within this window are selected, and their root mean square is calculated to represent the corresponding resampled EMG point to the Xsens point. This process ensures synchronization of the EMG signal with Xsens data, while simultaneously achieving smoothing and downsampling to the desired frequency. Note that this processing step influences the obtained results by affecting signal smoothness and the frequency and temporal resolutions. We chose the RMS approach because it provides a quantitative measure of muscle activation over short time windows by preserving the magnitude information of the raw EMG signal. The EMG signals were further normalized for each subject and each channel i.e. maximum values of each channel’s recorded EMG signal in the whole session of each subject were used to scale the data to the range [0, 1]. The corresponding motion data were scaled to the range $[-1, 1]$ to ensure consistent input ranges for subsequent analysis. For more comprehensive information about the dataset and study design, see^26^.

### Data setup

**Fig. 2:**
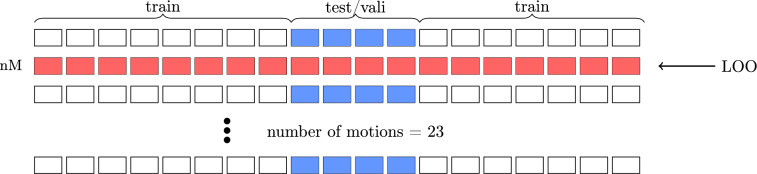
Dataset split for LSTM training. Rows represent the 23 different movements, and columns represent the 18 repetitions per movement. White indicates training data, blue indicates validation and test data, and red marks the left-out movement, also referred to as a new motion (nM), in the Leave-One-Out (LOO) setup. For each model, one of the 15 available movements is left out as nM to evaluate the model’s ability to generalize to unseen movements.

The dataset consists of 23 different movements (Table 1) ranging from simple, almost one-dimensional movements to more complex, everyday tasks. Each of these movements is systematically executed 18 times each by a group of five subjects, resulting in a rich set of data for analysis. We use 14 of these repetitions for the training dataset and 4 for the test and validation dataset (Fig. 2). The four repetitions for testing and validation were selected from the middle of the recording sequence to obtain representative samples, while avoiding only the earliest or latest repetitions. To account for individual differences between subjects, we implement a subject-specific modeling strategy, where the model is trained separately on each subject. To train the models, we adopt a Leave-One-Out (LOO) methodology. In this approach, all movements but one are utilized for training purposes, while the excluded movement is reserved as an independent new motion dataset. That leads us to have multiple models, each trained on different datasets and leaving one other movement out.

This strategy enhances the reliability of the evaluation of the model’s performance on previously unseen movements. We focused on 15 new motions, which is a slight reduction from the total 23 movements recorded. This adjustment is necessary due to the unavailability of specific movements for certain participants (Table 1). Thus, in order to maintain consistency across participants, only those movements that are uniformly available for all subjects are selected, yielding in a dataset of 15 new motions. Using the LOO approach, each model is trained on 22 of the 23 movements. However, due to errors in specific recordings for some subjects, testing on new motions was limited to these 15 uniformly available movements. The test dataset consists of two repetitions of each movement, excluding the new motion, thus including both simple and complex movements. Furthermore, the validation set utilized for early stopping includes one repetition of each movement, excluding the new motion, thus allowing for an evaluation process (Fig. 2). This leaves 14 repetitions of each movement allocated for training, again with each subject’s data excluding the new motion.

### LSTM model

The chosen Recurrent Neural Network (RNN) model employs Long Short-Term Memory (LSTM) layers for sequence modeling, making it well suited for time series analysis, physiological signal processing, and sequence based prediction tasks^44^. The model effectively learns sequential dependencies while overcoming challenges such as variable length inputs and overfitting, achieved through packed sequences, dropout regularization, and multiple LSTM layers. The models were hyperparameter-tuned for batch size, number of layers, number of nodes, and dropout rate using Optuna for OptKeras, with pruning enabled on an evolutionary sampler^45^. To minimize validation error, the optimization targeted the following parameters: batch size, number of layers, number of nodes, and dropout rate.

**Fig. 3:**
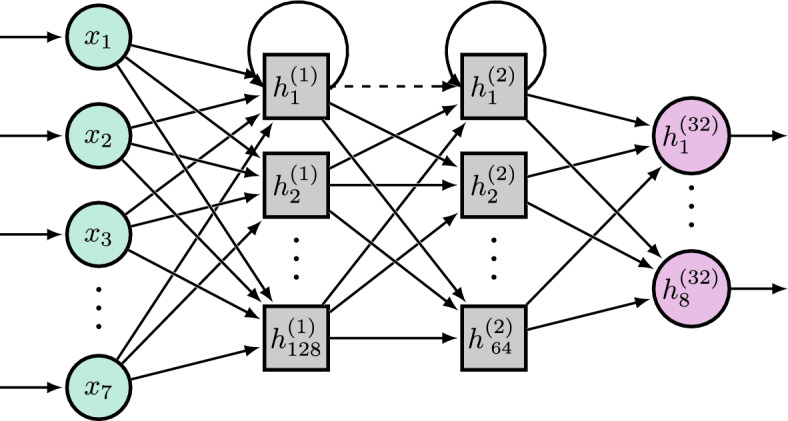
Recurrent Neural Network architecture with a linear movement input (green circles), two hidden LSTM (gray squares), and a linear muscle output (red circles) layer. The arrows that connect nodes $h_{1}^{(1)}$ and $h_{1}^{(2)}$ back to themselves are representative for all LSTM nodes. The dashed connection between the two LSTM layers indicated a dropout layer.

The architecture used in this work consists of two LSTM layers with hidden states with a size of 128 and 64 nodes, respectively (Fig. 3). The input sequences represent the end effector (EEF) states, specifically the position and orientation of the hand. Each LSTM layer processes input sequences in the format: batch size, sequence length, feature size, where the batch size is always 16. The feature size depends on the inclusion of elbow information, which either consists of 7 variables without elbow information or 8 with elbow information. The sequence length varies depending on the duration of each movement and differs between the two approaches: training on entire movements or segmented ones. To efficiently handle variable-length sequences, the model utilizes packed sequences, ensuring that padded time steps do not contribute to LSTM computations. The final LSTM output layer is processed through a fully connected (linear) layer that maps the learned features to the desired output space of 8 EMG signals.

### Evaluation metric

Assessing model performance requires robust evaluation metrics that quantify prediction accuracy and reliability. With *n* as the number of data we write

$$\begin{aligned} y_i&:\text {original data}\,,&\overline{y}&:\text {average of the original/predicted data}\,,\\ x_i&:\text {predicted data}\,,&\overline{x}&:\text {average of the original/predicted data}\,. \end{aligned}$$

A high mean squared error (MSE)

1 $$\begin{aligned} \textrm{MSE} = \frac{1}{n}\, \sum _{i=1}^{n} (y_i-x_i)^2 \end{aligned}$$

indicates significant deviations of predictions from actual values, suggesting poor model performance. Conversely, a low MSE reflects that predictions are close to actual values (measured as an absolute value), thereby indicating greater accuracy. Squaring the errors in MSE amplifies the effect of single larger errors on the final value. The squared correlation coefficient (r^2^)

2 $$\begin{aligned} \textrm{r} = \frac{\sum \limits _{i=1}^{n} (x_i-\overline{x}) (y_i-\overline{y})}{\sqrt{\sum \limits _{i=1}^{n} (x_i-\overline{x})^2 \cdot \sum \limits _{i=1}^{n} (y_i-\overline{y})^2}} \end{aligned}$$

measures the strength of the linear relationship between predicted and actual values but does not account for different magnitudes. A r^2^ value close to 1 denotes a strong correlation, where the predictions and actual values move together closely. In contrast, an r^2^ value approaching 0 signifies minimal to no linear relationship, indicating that the model’s predictions do not align well with true values. The coefficient of determination (R^2^)

3 $$\begin{aligned} \textrm{R}^{2} = 1-\frac{\sum \limits _{i=1}^{n} (y_i-x_i)^2}{\sum \limits _{i=1}^{n} (y_i-\overline{y})^2} \end{aligned}$$

measures the degree to which a model explains variability in the actual data. Values close to 1 imply that the model accounts for most of the variability, establishing it as a strong predictor. Conversely, an R^2^ value close to 0 indicates that the model explains very little variation, suggesting poor performance. A negative R^2^ is possible, implying that the model is less effective than simply predicting the mean of the data. The zero-line score (Z_s_)

4 $$\begin{aligned} {\textrm{Z}_{\textrm{s}}} = 100 \cdot \left( 1-\frac{\sum \limits _{i=1}^{n} (y_i-x_i)^2}{\sum \limits _{i=1}^{n} (y_i-0)^2}\right) \end{aligned}$$

is a variation of (R^2^), but instead of comparing the model’s performance to the variance around the mean $\overline{y}$, it compares the predicted data to a baseline where all values are zero. Consequently, the formula calculates how much better (or worse) the model does compared to simply predicting zero for all outputs. A score close to 100 means that the model performs very well, capturing almost all of the variance in the actual data. A score close to 0 suggests that the model is not much better than always predicting a zero signal. If the score is negative, it indicates that the model is performing worse than just using zero as a constant prediction, indicating it introduces output where there is very little in the original data. This metric is particularly useful when dealing with non-negative data with a lot of zero line such as the muscle activity, where a baseline of zero is the more natural reference point compared to the mean value as done in R^2^.

In summary, a reliable model exhibits a low MSE along with high r^2^, R^2^, and Z_s_ values indicating accurate predictions that follow actual data trends. Conversely, a high MSE combined with low r^2^, R^2^, and Z_s_ values signifies that the model lacks precision and does not effectively capture underlying data patterns.

### Statistics

For all comparisons, we use paired statistical tests to evaluate differences in model performance across subjects. We use parametric paired *t*-tests to assess whether the mean differences between conditions were significantly different from zero. For this, we first have to ensure that all our data is normally distributed. However, the zero-line score (Z_s_) used here, similar to other correlation coefficients, is always skewed to the left. This is because of the maximum value at 100 for Z_s_ (or 1 for r), e.g. a change from 80 to 90 Z_s_ describes a significantly higher difference than a decrease from 80 to 70 Z_s_. Thus, to account for the skewness and its non-linear effect in our performance measure, we apply Fisher’s z-transformation using hyperbolic arctangent to our zero-line score (Z_s_) to improve the approximation to normality. In addition, we test the assumption of normally distributed differences using the Shapiro–Wilk test, which is suitable for small sample sizes. Only in one case, the normality assumption with statistical significance at $p<0.05$ is violated (Supplementary Table S4), and a non-parametric Wilcoxon signed-rank test is used instead.

## Results

In this section, we present the evaluation of our LSTM model (Section LSTM model), which is designed to predict upper limb muscle activity based on the position and orientation of the end effector (EEF). Our dataset includes 23 distinct movements, which range in complexity from simple to everyday tasks, as detailed in Table 1. Data were collected from five subjects. To account for inter-subject variability, we employ a subject-specific modeling approach, training an individual model for each participant. For the evaluation process, we implemented a standard train-test split alongside a Leave-One-Out (LOO) approach (Fig. 2). In this framework, all movements except one movement are utilized for training, while the excluded movement is treated as an unseen new motion dataset designated for further testing. This strategy facilitates an assessment of the model’s ability to generalize to completely new motions, a particularly demanding task due to the inherent variability in movement generation. Out of a total of 23 movements, we identify 15 new motions that had not been represented in the training phase, as not all movements were performed by each subject (Table 1). These new motions represent a particularly difficult test case, as the model has never encountered this specific type of motion during training. Successfully predicting muscle activity for these movements demonstrates the model’s ability to infer patterns and extrapolate to unseen movement dynamics. A detailed description of the training procedure is given in Section Data setup.

**Fig. 4:**
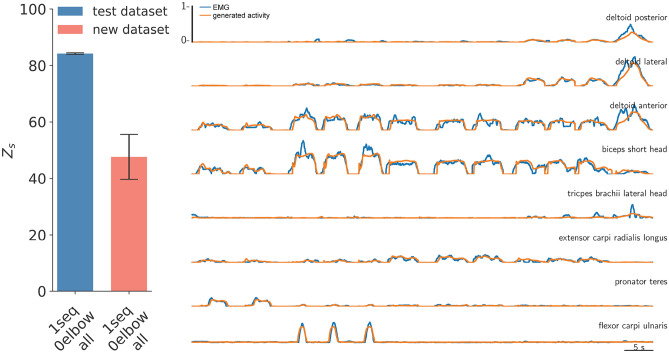
Performance of the LSTM model trained on entire movement sequences (1seq), without elbow information (0elbow), evaluated using the Z_s_ score across all data types (all). The bar chart displays the model’s performance on the test and new motion datasets with a median across five subject-specific models, all movement types, over five repetitions, with median absolute deviation between subjects as whiskers. On the right, an example prediction from the test dataset illustrates the recorded muscle activity for all eight EMG channels (blue), alongside the model generated muscle activity (orange).

The LSTM model is trained utilizing entire movement sequences, with each movement treated as an individual sequence for training purposes. The model demonstrates remarkable efficiency on the test dataset, achieving a median accuracy Z_s_ score of 84 (Fig. 4) (MSE $=0.0028$, r^2^ $=0.79$ and R^2^ $=0.79$, cf. Table 2). However, when the model is tested on a new dataset containing entirely unseen motion sequences, there is a decline in performance with prediction falling to a median Z_s_ score of 46 (MSE $=0.0050$, r^2^ $= 0.54$, and R^2^ $= 0.07$, cf. Table 2) which is roughly half of the accuracy observed on the test dataset. To provide a more intuitive understanding of these results, Fig. 4 also shows a direct comparison of the recorded and predicted muscle activity signals for a subset of the test dataset. Further examples of side-by-side comparisons between predicted and recorded muscle activity are provided in Supplementary Figure S1 for the test dataset, and in Supplementary Figure S2, S3, and S4 for the new dataset. The predicted signals closely matches the original signals, particularly in terms of timing and amplitude. Nevertheless, finer details such as complex peak structures are not always accurately captured. This discrepancy is expected, as neural networks tend to generalize patterns while smoothing out minute variations inherent in highly dynamic signals.

### Prediction for unseen movements

**Fig. 5:**
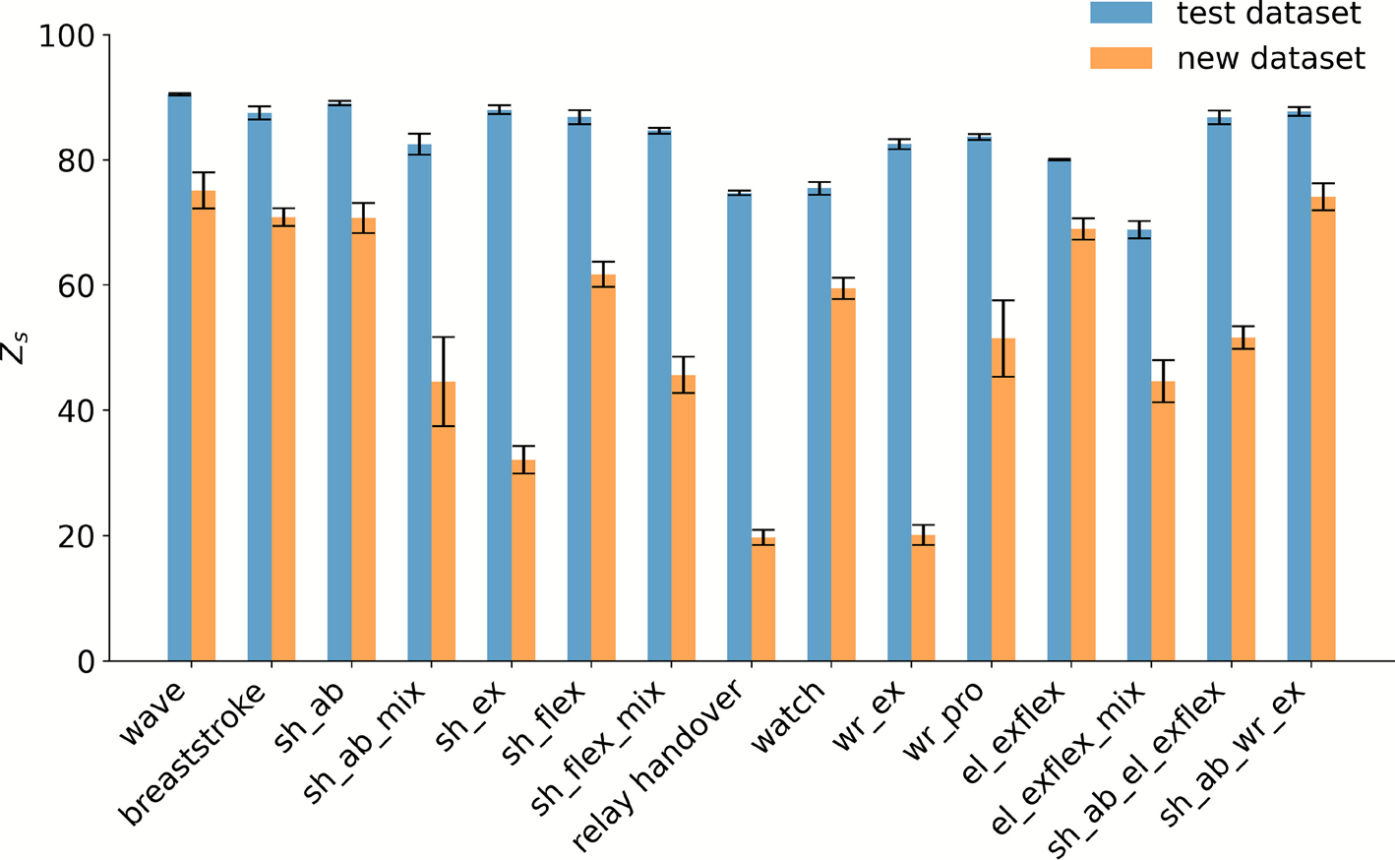
Performance of the LSTM model trained on entire movement sequences (1seq), without elbow information (0elbow), evaluated using the Z_s_ score across all data types (all). The bar chart displays the model’s median performance on the test and new motion datasets for all movement types, each evaluated over five repetitions, with median absolute deviation as whiskers. The results highlight a subject with a better predictive performance. The *x*-axis reports the individual movement types, written out as: waving gestures, breaststroke, shoulder abduction, shoulder abduction (mix), shoulder extension, shoulder flexion, shoulder flexion (mix), relay handover, reading a watch, wrist extension, wrist pronation, elbow flexion, elbow flexion (mix), shoulder abduction with simultaneous elbow flexion, and shoulder abduction with simultaneous wrist extension. Simple movements are abbreviated using a joint–motion schema: (sh) denotes shoulder, (el) elbow, and (wr) wrist, followed by the motion direction (ab) for abduction, (ex) for extension, (flex) for flexion, and (pro) for pronation.

**Fig. 6:**
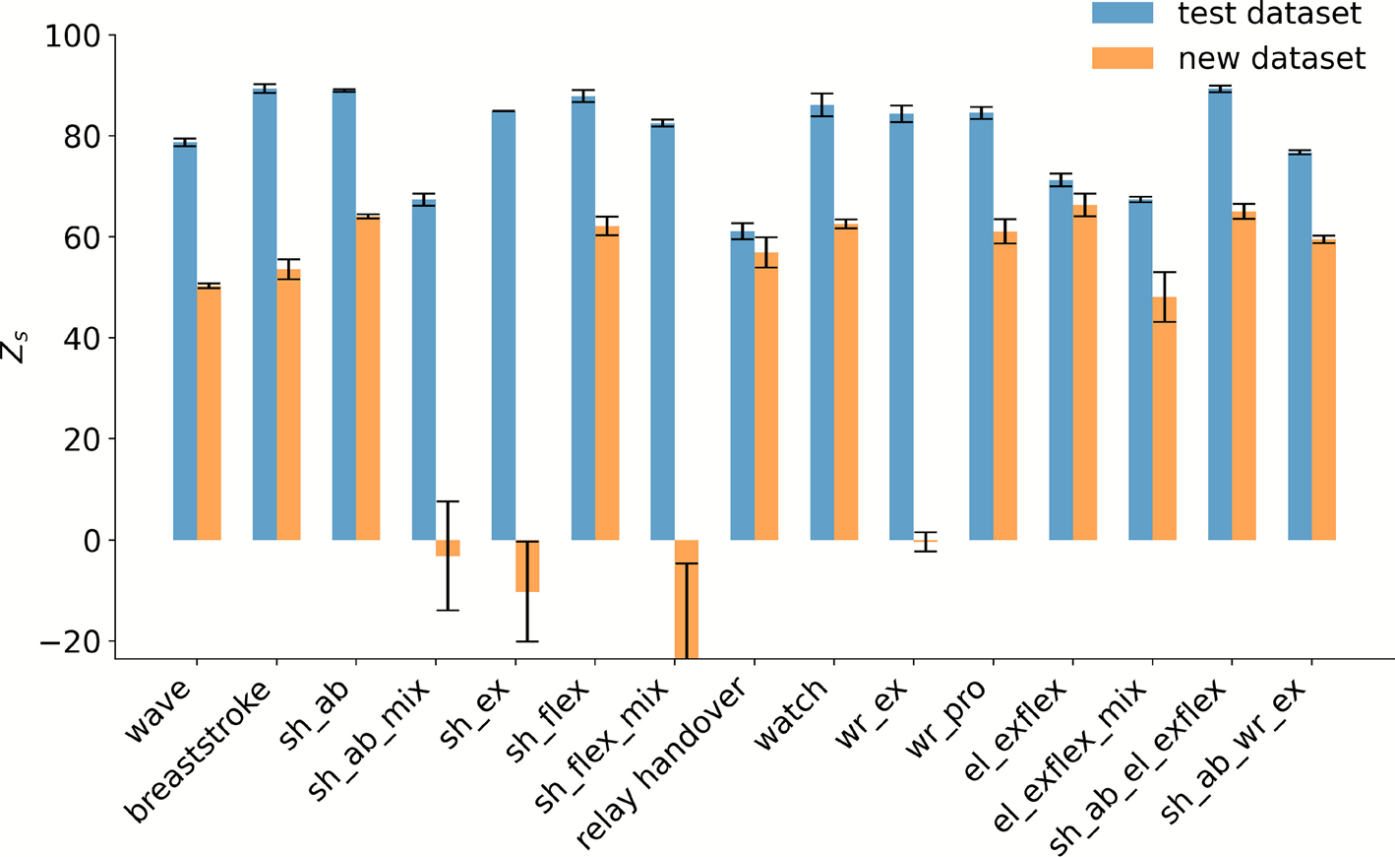
Performance of the LSTM model trained on entire movement sequences (1seq), without elbow information (0elbow), evaluated using the Z_s_ score across all data types (all). The bar chart displays the model’s median performance on the test and new motion datasets for all movement types, each evaluated over five repetitions, with a median absolute deviation as whiskers. The results highlight a subject with a lower predictive performance. The x-axis reports the individual movement types, written out as: waving gestures, breaststroke, shoulder abduction, shoulder abduction (mix), shoulder extension, shoulder flexion, shoulder flexion (mix), relay handover, reading a watch, wrist extension, wrist pronation, elbow flexion, elbow flexion (mix), shoulder abduction with simultaneous elbow flexion, and shoulder abduction with simultaneous wrist extension. Simple movements are abbreviated using a joint–motion schema: (sh) denotes shoulder, (el) elbow, and (wr) wrist, followed by the motion direction (ab) for abduction, (ex) for extension, (flex) for flexion, and (pro) for pronation.

In this section, we take a closer look at the predictions for new motions and perform a more detailed analysis. To this end, we present the predictions for each movement type separately. In accordance with the finding in Fig. 4, the test dataset maintains a stable performance throughout all motions with median scores around 80. However, predictions for the new motion dataset reveal lower median scores and substantial variability both between subjects within specific movement types and also between the different movement types themselves. For instance, one subject’s scores fluctuate between approximately 70 and 20 (Fig. 5) while another’s range from 65 to -22 (Fig. 6). Furthermore, some subjects’ movements are predicted with higher accuracy than others. For the latter subject with the lower predictive accuracy, four movement types stand out with particularly poor performance: shoulder flexion mix, shoulder extension, shoulder abduction mix, and wrist extension. Interestingly, these movements are all categorized as simple movements. In contrast, some of the more complex movements, such as the breaststroke and reading a watch motion, demonstrate more robust predictive accuracy for all subjects.

**Fig. 7:**
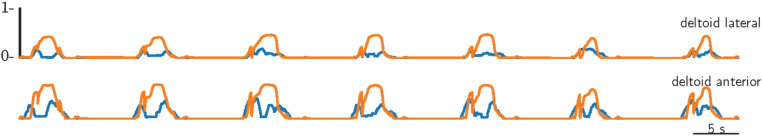
Prediction for the shoulder flexion mix new motion illustrating the recorded muscle activity (blue) alongside the model generated muscle activity (orange), showing only signal of the relevant muscles: lateral and anterior deltoid. The results highlight a subject with a lower predictive performance.

**Fig. 8:**
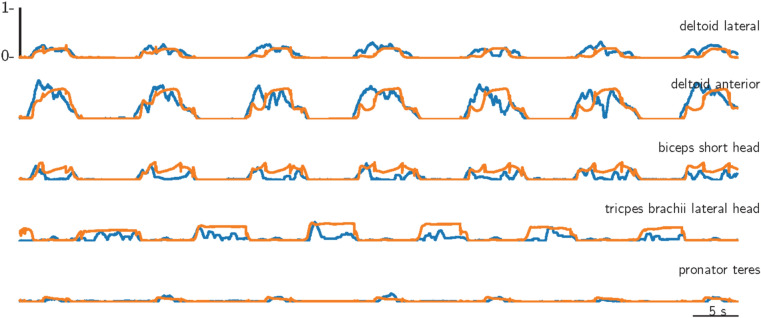
Prediction for reading a watch new motion illustrating the recorded muscle activity (blue) alongside the model generated muscle activity (orange), showing only the relevant muscles: lateral and anterior deltoid, biceps, triceps, and pronator teres. The results highlight a subject with a lower predictive performance.

To provide a more intuitive understanding of these results, we include a detailed side-by-side comparison of the recorded and predicted signals. Specifically, we focus on the subject with a lower predicted accuracy and select the movement type with the weakest performance, i.e. shoulder flexion mix (Fig. 7). A clear discrepancy can be seen in the deltoid lateral and anterior activity (simple movement), where the predicted signal overshoots the recorded signal. While the timing remains accurate, the amplitude is noticeably off, which is a typical characteristic of poorly predicted new motion types and the primary reason for the lower scores. For comparison, we also show reading a watch from the same subject, see Fig. 8. This movement involves several muscles (complex movement), and the timing again aligns well with the recorded data. In addition, the amplitude is captured more accurately, further highlighting the variability in predictive performance across different movements.

### Redundancy in arm kinematics: The impact of the swivel angle

In the following section, we examine whether incorporating more precise positional information about arm configuration enhances prediction accuracy. The human upper limb is a redundant system, possessing more degrees of freedom (DOF) than are strictly necessary to position and orient the end effector (EEF) in space. Specifically, the arm has 7 DOF, 3 for the shoulder, 1 for the elbow, and 3 for the wrist, not including further scapular DOFs, whereas the spatial positioning and orientation of the EEF require only 6 coordinates in total (3 each). Thus, for a given EEF state, there are an infinite number of possible arm configurations, which is a well-known problem in inverse kinematics. Redundancy in arm kinematics is also a central topic in motor control, and various theories have been proposed to explain how the nervous system manages this excess of DOF. In undisturbed reaching movements, arm configurations are typically executed with high consistency, resulting in minimal variation in both arm configuration and swivel angle across trials^46^. Therefore, we do not expect substantial performance improvements from the inclusion of elbow information for most movements in our study. Nevertheless, incorporating this information contributes to the robustness of the model, particularly under perturbed or less constrained conditions.

**Fig. 9:**
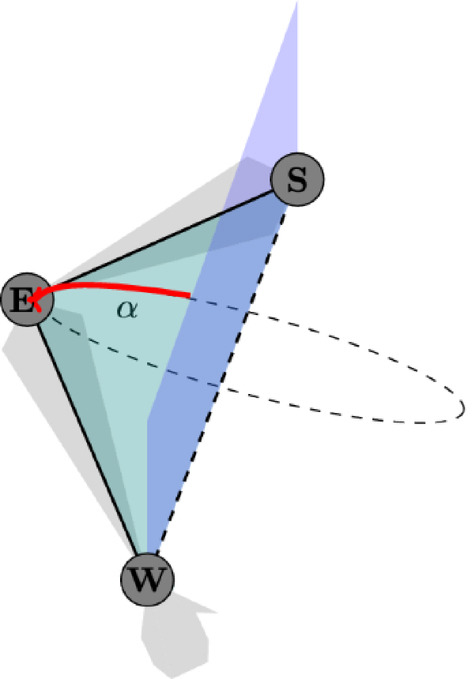
Swivel angle $\alpha$ determined by the angle between arm plane $\mathbf {n_A}$ (green) and reference plane $\mathbf {n_R}$ (blue). Shoulder (S) and wrist (W) are on both planes, and the arm plane is defined by the elbow (E), while the reference plane is defined with the reference vector (0, 0, 1). The grey areas represent the human arm.

One way to parameterize this redundancy is through the swivel angle, which represents the rotational degree of freedom at the elbow. The swivel angle is defined^28,29^ as the angle between the plane of the arm formed by the shoulder, elbow, and wrist, and a reference plane given by the shoulder, wrist, and the $z-$axis (0, 0, 1) (see Fig. 9). This angle is calculated from the normal vectors of each plane, as illustrated in Equation 5. Thus, it is zero when the elbow is exactly in the reference plane, i.e. the elbow is exactly below the shoulder wrist line.

5 $$\begin{aligned} \cos (\alpha ) = \frac{\mathbf {n_A} \cdot \mathbf {n_R}}{\Vert \mathbf {n_A}\Vert \Vert \mathbf {n_R}\Vert }\,, \end{aligned}$$

While this definition of the swivel angle can be used for most movements where the arm moves close around the horizontal $xy-$plane it becomes ill-defined when the wrist is located exactly below (or above) the shoulder. Unfortunately, this is the case for the resting position of all our motions studied in this work. Here, the shoulder-wrist vector is nearly parallel to the $z-$ axis and, therefore, the associated span cannot be used to form the reference plane. An improved definition called the elbow swivel angle (ESA)^47^ incorporates a moving help vector instead of the $z-$axis to ensure a well-defined definition of the reference plane throughout the whole motion. For the resting position, we choose the $x-$axis, which is perpendicular to the shoulder-wrist vector, and let it rotate with the executed movement of the arm. Thus, the reference plane is well-defined at every timestamp of all motions. A similar problem happens if the arm is fully straightened so that the elbow position is on the shoulder-wrist line, making the definition of the corresponding arm plane ill-defined as well. Again, to a lesser extent, this happens for the resting position and a few simple movements like shoulder extension and abduction. We control this numerical inaccuracy by evaluating the distance between elbow and shoulder-wrist line.

A pure elbow rotational movement without altering the position or orientation of the EEF, still significantly influences the orientation of the arm itself. Consequently, for any defined EEF state, the elbow can adopt multiple orientations due to the redundancy in human arm kinematics. Therefore, in addition to the EEF as an input to predict muscle activity, the swivel angle might be crucial because the muscle activation pattern depends on the joint configuration (not just the EEF), i.e. all 7 DOF of the arm movement are necessary. Different elbow information (swivel angles) can lead to different moments in the arm, which will also affect muscle force generation. Thus, understanding and incorporating the swivel angle could improve predictive models of muscle activation in relation to arm movements. In the following, we refer to models that include the elbow information (swivel angles) as 1*elbow*, and those that do not include them as 0*elbow*.

**Fig. 10:**
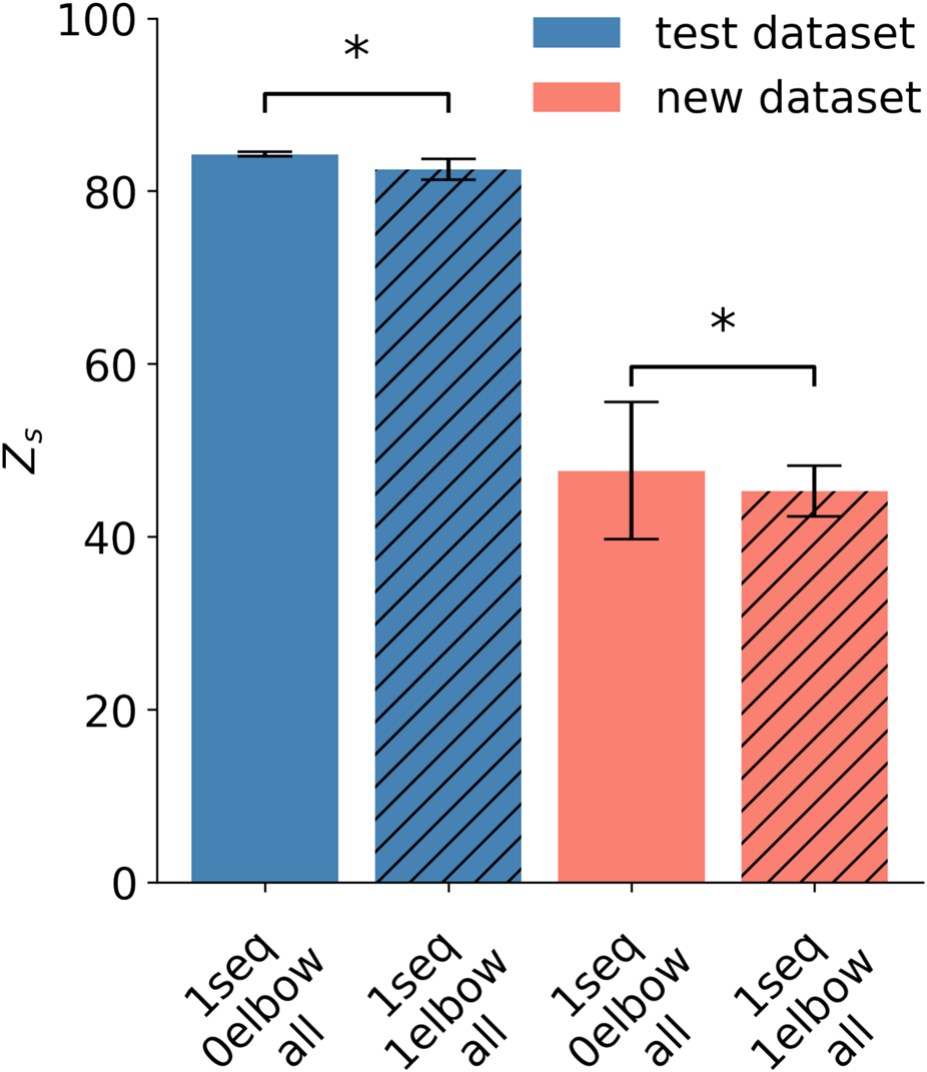
Performance of the LSTM model trained on entire movement sequences (1seq), without elbow information (0elbow), and with elbow information (1elbow and hatched bar) evaluated using the Z_s_ score across all data types (all). The bar chart displays the model’s performance on the test and new motion datasets, with a median across five subject-specific models and movement types, each evaluated over five repetitions, with median absolute deviation between subjects as whiskers. An asterisk (*) indicates a statistically significant difference ($p < 0.05$) based on a paired *t*-test.

To assess the effect of incorporating the swivel angle, we trained the LSTM model with and without this additional input. Our results indicate that including the swivel angle does not improve the model’s performance. In fact, both the test and new motion datasets show slightly lower scores within the deviation of our model when elbow information (swivel angle) is included compared to when it is omitted (Fig. 10), with scores of Z_s_ = 81.79 (MSE $= 0.0032$, r^2^ $= 0.76$, and R^2^ $= 0.74$, cf. Table 2) for the test dataset, and Z_s_ = 41.85 (MSE $= 0.0052$, r^2^ $= 0.54$ and R^2^ $= -0.03$, cf. Table 2) for the new dataset in the 1*elbow* condition. This observation can be further supported by a paired *t*-test indicating a significant decrease in performance with additional information ($t = -3.97$, $p = 0.016$, $d=1.78$) for the test dataset and for the new dataset ($t = -3.49$, $p = 0.025$, $d=1.56$), indicating a very large effect. These results suggest that adding the extra elbow information systematically reduced model performance across all subjects.

As expected, the inclusion of the swivel angle does not lead to a significant difference in model performance. To better understand this result, we examine the issue with an additional mathematical perspective. For simple movements, such as shoulder abduction with an outstretched arm, the swivel angle has little impact on the overall position of the arm (and is even ill-defined). In this scenario, the upper limb moves away from the body in the frontal plane, while the elbow remains fully extended. This can be explained by considering the shoulder-to-hand and elbow-to-shoulder vectors. Since these vectors remain almost parallel throughout the movement, any rotation around this axis does not affect the final position of the EEF. Likewise, during a flexion and extension of the elbow constrained in one plane, the significance of the swivel angle is limited. In this context, while the shoulder and elbow maintain in a fixed position, the forearm moves in a fixed circular movement around the elbow joint. This single plane movement implies that the swivel angle is constant and thus has a negligible effect here.

Conversely, for more complex (out of plane) movements, such as waving or the arm movements of the breaststroke, the swivel angle plays a role. For instance, in the breaststroke, the arm undergoes a coordinated pattern of flexion, abduction, and rotation at the shoulder, alongside elbow flexion and extension. Unlike simple single-joint movements, the trajectory of the hand in these more complex movements is affected by both the EEF and the swivel angle. If the swivel angle is not properly controlled, multiple shoulder and elbow configurations can result in identical EEF positions, thereby creating potential ambiguity. In this context, the swivel angle is essential to fully resolve the three-dimensional orientation of the arm.

**Fig. 11:**
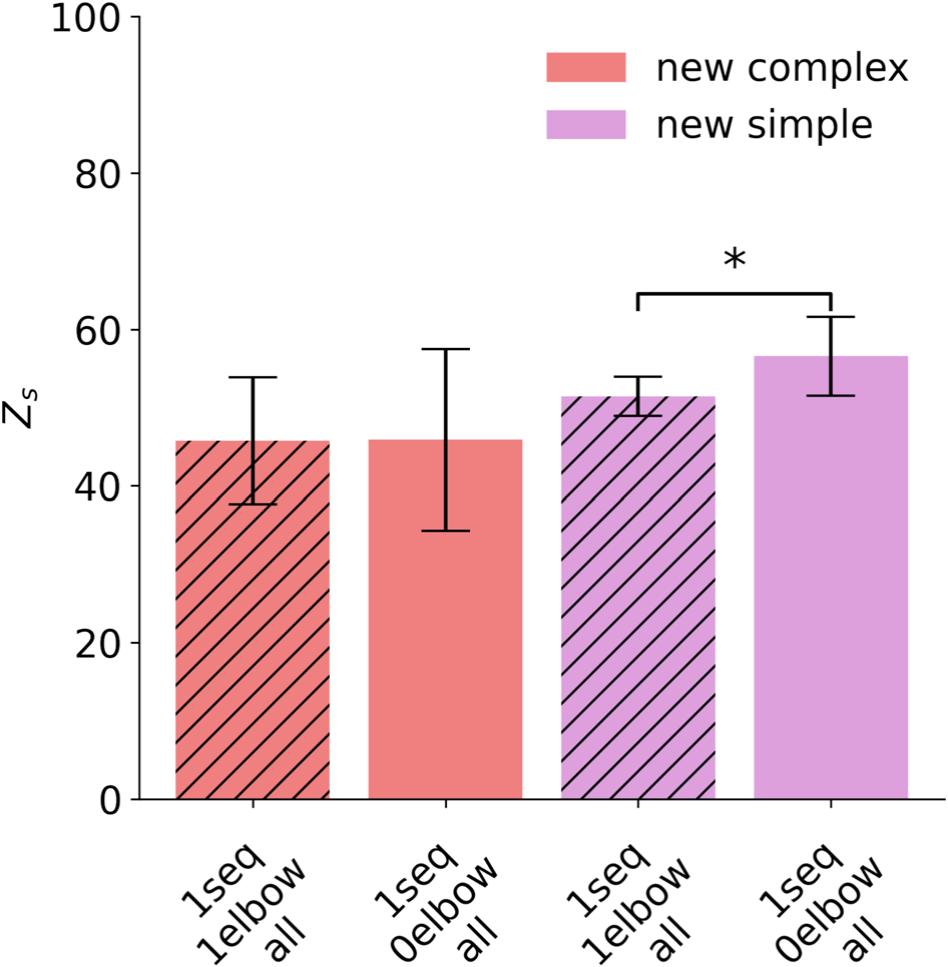
Performance of the LSTM model trained on entire movement sequences (1seq), without elbow information (0elbow), and with elbow information (1elbow and hatched bar), evaluated using the Z_s_ score across all data types (all). The bar chart displays the model’s performance on the new motion datasets, with a median across five subject-specific models and movement types, each evaluated over five repetitions, with median absolute deviation between subjects as whiskers. Movements considered with crucial elbow information are wave, breaststroke, and reading a watch. Movements with no crucial elbow information are shoulder abduction, shoulder flexion, wrist pronation, and elbow extension flexion. An asterisk (*) indicates a statistically significant difference ($p < 0.05$) based on a paired *t*-test.

Thus, while the swivel angle is often irrelevant for simple movements involving a single-joint, it has more influence for complex, multi-joint movements, where multiple degrees of freedom influence the overall movement. To visualize the potential impact of including elbow information, we further divided the movement types into two groups: First, movements that are likely to benefit from swivel angle information: Waving, breaststroke, and reading a watch. Second, movements unlikely to be affected by the swivel angle: Shoulder abduction, shoulder flexion, wrist pronation, and elbow extension flexion (Fig. 11, Supplementary Table S1). We then subsequently analyzed model performance within these two categories. Our findings suggest a slight advantage in performance for movements that are dependent on elbow information when the swivel angle is considered, due to the reduced deviation. On the contrary, movements that are less influenced by elbow information exhibit slightly better performance when this data is excluded reaching higher scores, particularly with Z_s_ = 56.57 and R^2^ $= 0.244$ (cf. Supplementary Table S1) for new movements. This observation is further supported by the results of a paired *t*-test ($t = -3.2$, $p = 0.032$, $d=1.43$), indicating a significant difference between the two groups. Notably, the R^2^ values are generally low for new motions (see Section Overview for a more detailed discussion); however, they tend to be comparatively higher in conditions without elbow information, especially for new simple and complex movements. Nevertheless, high deviations in both cases limit the ability to draw definitive conclusions. Although observable trends exist, further inquiry is necessary to determine whether the swivel angle consistently improves movement prediction efficacy.

### Movement complexity

When developing predictive neural network models, the complexity of the training data is a critical factor influencing model performance. A key consideration is whether a model achieves better generalization by being trained on a diverse set of movements or by specializing in either simpler or more complex movements. To explore this, we compared three LSTM models: one trained on all movement types, one dedicated to simple movements predominantly characterized by near one-dimensional movements, and a third focused on complex movements involving multiple joints and exhibiting greater degrees of freedom. Briefly, the results revealed that the LSTM model trained on all movement types achieved a score of 84 on the test dataset and 46 on a separate dataset of new types of motions.

**Fig. 12:**
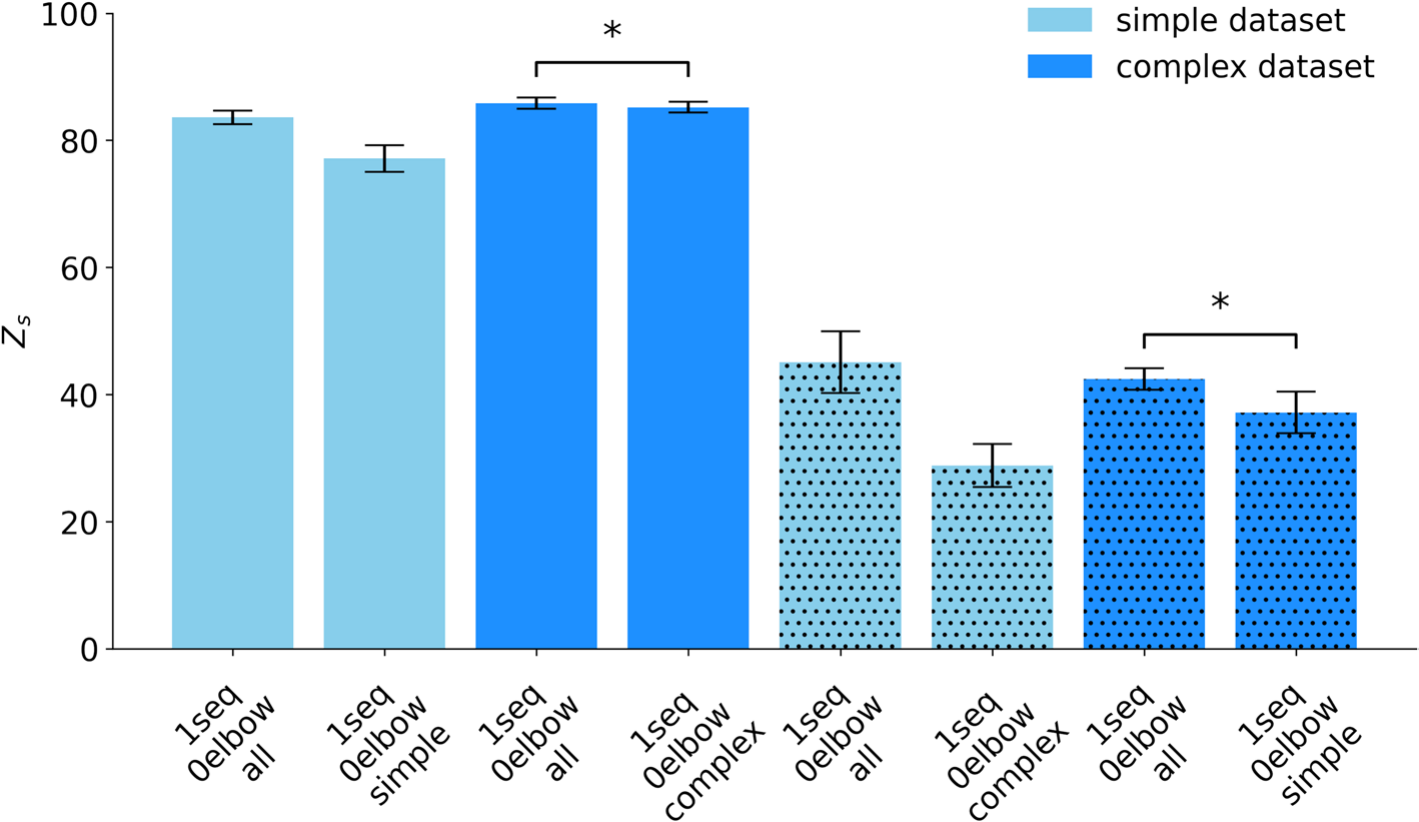
Average performance of the LSTM model trained on entire movement sequences (1seq), without elbow information (0elbow), evaluated using the Z_s_ score across all data types (all), simple data types (simple), and complex data types (complex). The bar chart displays the model’s performance on simple movement exclusive test dataset and complex movement exclusive test dataset (different shades of blue). The dot pattern indicates where either new simple motions or new complex motions are used. Results are the median across five subject-specific models and multiple movement types, with each evaluated over five repetitions. The whiskers present the median absolute deviations. An asterisk (*) indicates a statistically significant difference ($p < 0.05$) based on a paired *t*-test.

To further evaluate the performance of the simple and complex models, we introduce two new test sets: a simple movement exclusive test dataset, containing only simple movements, and a complex movement exclusive test dataset, containing only complex movements. This distinction is essential, as the original test dataset included both simple and complex movements, posing challenges for models trained exclusively on one category due to the presence of unfamiliar, unseen movement types. Each model performs well when tested within its own category. Specifically, the simple movement trained model performs well on the simple movement exclusive test dataset while the complex movement trained model excelled on the complex movement exclusive test dataset, reaching Z_s_ scores of 77.16 and 85.21, respectively (Fig. 12 and Supplementary Table S2). This indicates that exposure to more complex movements may improve generalization across varied movement scenarios. Note that the base model is trained on all data, while the complex and simple models can only use the data of roughly half of the movement types each. Although limited to roughly half of the movement types, the complex model still achieves a median performance comparable to that of the all model. Nonetheless, paired *t*-test results indicate a significant difference between the two ($t = 3.46$, $p = 0.026$, $d=1.55$) with the all model being significantly better. A notable decline in performance is observed when each model is assessed in the opposite category, when predicting new motions from the other model’s category. The performance of the simple and complex models lagged behind the all model. For a better comparison, the all model also predicts either the simple new motion or complex new motions, indicated by a distinct dot pattern (Fig. 12 and Supplementary Table S2). These results suggest that training on a wider variety of movements facilitates better generalization, probably due to the model’s ability to learn a richer representation of movement patterns. This effect is further supported by a significant difference between the all model tested on the complex dataset and the simple-trained model tested on the complex dataset ($t = 2.98$, $p = 0.043$, $d=1.31$), showing a significantly better performance trained on the all model.

### Decomposition of movement sequences

**Fig. 13:**
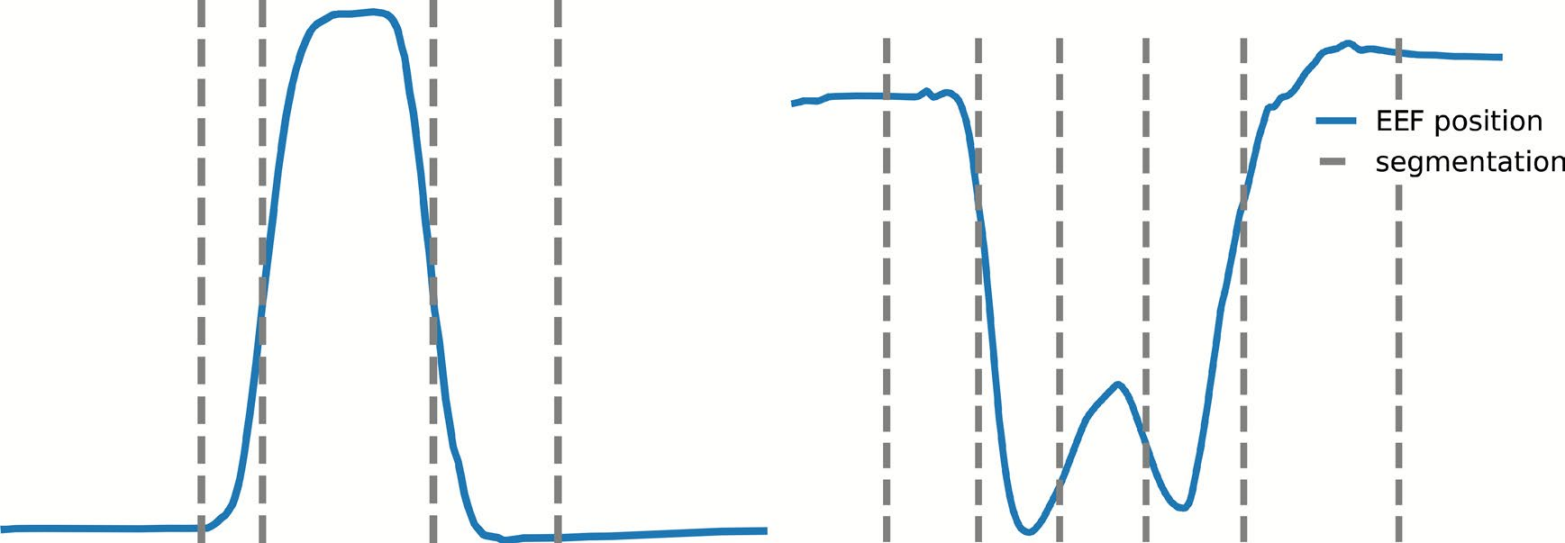
Illustration of two exemplary movements: (left) shoulder flexion with simultaneous elbow extension and flexion, represented by the EEF ($y-$position) over time, and (right) reading a watch, represented by the EEF ($x-$position) over time. In both cases, the movement trajectory is shown by the blue line. Gray dashed lines indicate segmentation points, representing both the inflection points and the velocity-based start/stop thresholds that define the beginning and end of each segment.

So far, we have categorized movements into simple and complex movements (see Table 1). We now want to examine the potential benefits of decomposing these movements into shorter sub-sequences. As previously mentioned (see Introduction 2), Krebs et al. support a segmentation approach based on submovements that utilized a bell-shaped velocity profile^33^. While this is similar to our unsegmented simple movements, it does not apply to more complex ones. We decided to use a similar approach, but instead of a bell-shaped velocity profile, we implemented a bell-shaped acceleration profile. This decision allows for finer segmentation, even in simple movements, resulting in a greater number of subsequences. This choice is further motivated by the physiological relationship between acceleration and underlying muscle forces. This decomposition is accomplished through a segmentation method that focuses on identifying inflection points within the movement trajectory. Inflection points are mathematically defined as the locations where the second derivative of the trajectory changes sign, indicating structural transitions in the motion. By segmenting the movements at these inflection points (Fig. 13), we aim to generate sub-sequences that exhibit greater kinematic homogeneity. This could potentially reduce variability within each segment. We hypothesize that this finer segmentation may enhance model performance compared to using full movement sequences if the model now draws a better understanding between the segmented kinematic sequences and their corresponding muscle activity. Beyond performance considerations, segmentation is also conceptually linked to competing theories of motor control. One perspective views complex movements as being composed of simpler primitives^30,31^, which segmentation at inflection points directly tests. The alternative perspective emphasizes that neuromuscular coordination emerges from global, multi-joint patterns^34,35^. By comparing intact sequences (1*seq*) with segmented sequences (*nseq*), our analysis provides a framework to contrast these two views.

**Fig. 14:**
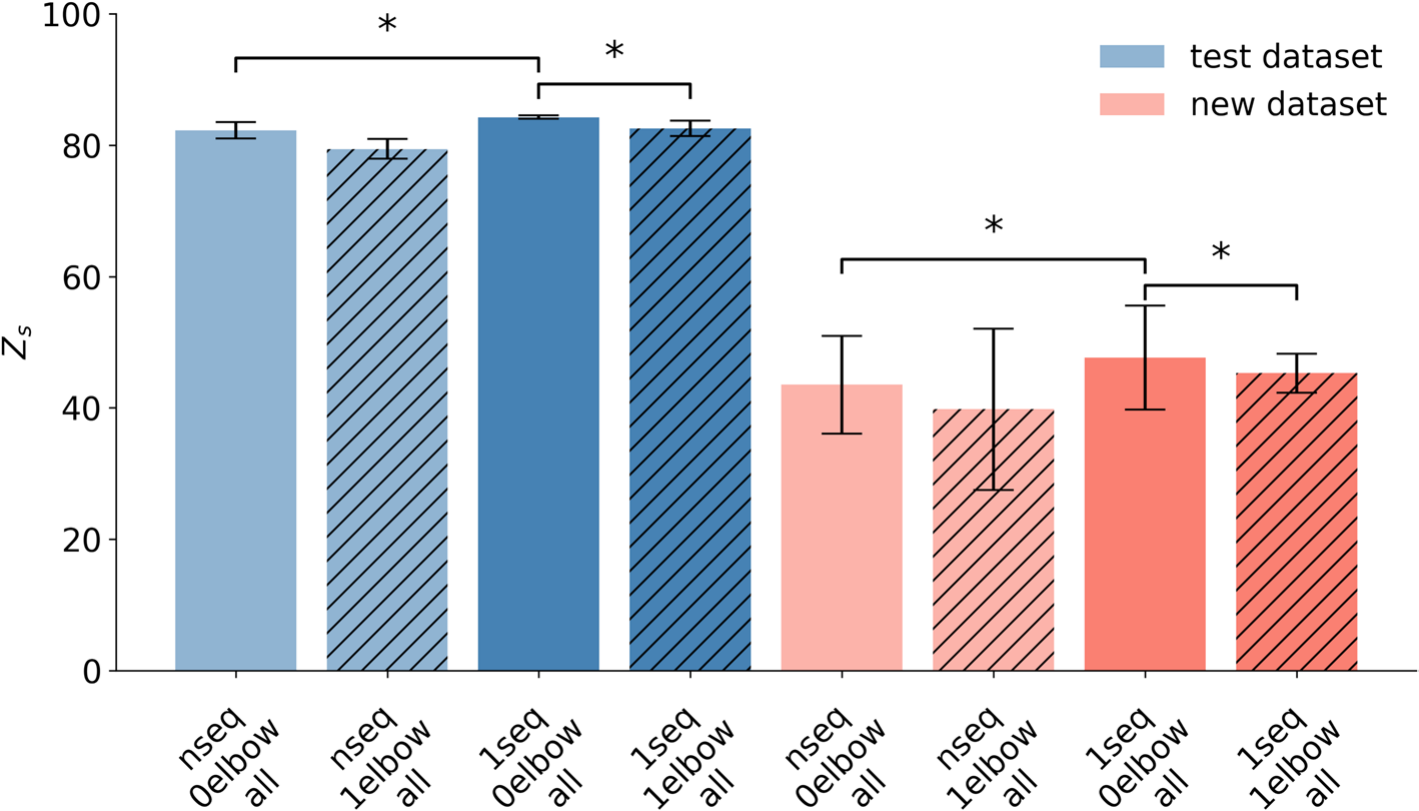
Performance of the LSTM model trained on entire movement sequences (1seq), and sub-sequences (nseq), without elbow information (0elbow), and with elbow information (1elbow and hatched bar), evaluated using the Z_s_ score across all data types (all). Results are the median of five subject-specific models and multiple movement types, with each evaluated over five repetitions. The whiskers present the median absolute deviations. An asterisk (*) indicates a statistically significant difference ($p < 0.05$) based on a paired *t*-test.

To evaluate this, we compare two approaches: one where the entire movement sequence (1*seq*) is used without segmentation, i.e. the previous model (1*seq*), and another where segmentation at the inflection points is applied, referred to as (*nseq*). Here, *n* denotes the number of segments obtained from the inflection points of a given movement. Simple movements typically yield around three segments, whereas complex movements can result in a higher number of segments, as illustrated by the varying number of gray dashed lines in Fig. 13. Both the 1*seq* and *nseq* models are based on the same network architecture described in Section LSTM model. The distinction lies in the training and evaluation data: the 1*seq* model is trained and tested on entire movement sequences, while the *nseq* model is trained and tested on segmented sub-sequences. Consequently, the main difference concerns the input sequence length. Contrary to our expectations, the findings revealed no significant performance enhancement associated with the sub-sequence decomposition. The model trained on entire movement sequence (1*seq*) has a significantly higher performance than the segmented movement when no elbow information is included for the test and new motion dataset shown by a paired *t*-test ($t = -3.28, p = 0.030$, $d=1.47$) and ($t = -4.51, p = 0.011$, $d=2.02$). The median performance metrics for the *nseq* approach are close to those of the 1*seq* method as illustrated in Fig. 14 and in Table 2. As noted in Section Redundancy in arm kinematics: The impact of the swivel angle, for new motions the model trained without elbow information consistently exhibits higher R^2^ values, independent of the segmentation approach Supplementary Table S3.

### Overview

For a comprehensive evaluation, we compile performance metrics across all conditions in Table 2, which shows a detailed comparison of how the different conditions compare to each other. Additionally, we include various scoring metrics to facilitate cross-study comparisons and to provide a broader perspective on model performance.

**Table 2:** Overview of the LSTM model performance for the test and new motion dataset under various conditions: entire movement sequences (1seq), sub-sequences (nseq), without elbow information (0elbow), and with elbow information (1elbow). The performance is evaluated by the zero-line score Z_s_, mean squared error MSE, the squared correlation coefficient r^2^, and the coefficient of determination R^2^.

	score	1*seq*, 0*elbow*	1*seq*, 1*elbow*	*nseq*, 0*elbow*	*nseq*, 1*elbow*
test prediction	Z_s_	**84.49**	81.79	82.67	80.50
	MSE	0.0028	0.0032	0.0031	0.0035
	r^2^	0.79	0.76	0.76	0.74
	R^2^	0.78	0.74	0.75	0.72
new prediction	Z_s_	**46.16**	41.85	43.87	41.07
	MSE	0.0050	0.0052	0.0050	0.0051
	r^2^	0.54	0.54	0.52	0.51
	R^2^	0.07	-0.03	0.03	-0.003

Analysis of the test dataset indicates strong predictive performance for all four metrics: the zero-line score Z_s_, mean squared error MSE, the squared correlation coefficient r^2^, and the coefficient of determination R^2^. As expected, the new dataset consisting of unseen data exhibits lower scores. Notably, the R^2^ appears surprisingly low, with the only score that has some negative values. We note that Z_s_ differs from R^2^ in the denominator by comparing the signal to zero, which is the natural baseline for electromyography signals, rather than to the mean $\overline{y}$ of the signals. In this particular case of this study, where the signal is strictly non-negative and has a resting point at zero, Z_s_ proves to be more suitable for evaluation. This discrepancy in metrics is not only observed for Z_s_ but also extends to MSE and r^2^. The lower values for R^2^ are mainly due to its greater sensitivity to overshooting or low value predictions, whereas MSE treats over- and under-predictions equally. This phenomenon is evident in new motions, where the amplitudes are not accurately predicted, as illustrated in Fig. 7 and Supplementary Figure S3. These new motions exhibit a tendency to result in overshooting predictions, which may explain the observed decrease in R^2^ scores. Notably, models trained with elbow information perform in general significantly worse than without; these models tend to exhibit even lower R^2^ values for new motions than those trained without. Beyond visual comparison, this underscores the importance of considering multiple evaluation metrics in parallel, since each captures distinct error characteristics and provides complementary insights into model performance.

## Discussion

Our primary objective is to unravel the relationship between upper limb kinematic data and corresponding muscle activity. The study focuses on training a predictive model, specifically a Long Short-Term Memory (LSTM) network, using kinematic end effector (EEF) data to estimate muscle activity. LSTM networks are highly effective for modeling sequential dependencies in EMG data, and typically outperform feedforward and convolutional neural networks for this task. Feedforward architectures lack intrinsic mechanisms for capturing temporal dynamics, whereas CNNs can extract local temporal or spatial patterns but primarily model short-term dependencies^26^. Transformer-based architectures have recently demonstrated superior performance in time-series prediction, due to their self-attention mechanism, which efficiently captures long-range temporal dependencies^48^. In our study, the sequence lengths of the segmented reaching movements were relatively small, making LSTMs a suitable and efficient choice. Moreover, the recurrent dynamics of LSTMs conceptually align with temporal integration processes in biological motor control, supporting their physiological plausibility. Future work could explore hybrid or attention-augmented recurrent architectures that combine the interpretability of recurrent networks with the representational power of Transformers. Previous research suggests that LSTMs effectively capture temporal dependencies and that EEF data provides a more efficient representation compared to individual joint angles^26^. This study utilized subject-specific models instead of a generalized model due to the high inter-subject variability observed in surface electromyography (sEMG) signals, deriving from differences in anatomy, electrode placement, and subcutaneous tissue properties^49–54^. General models often show reduced predictive performance when applied to unseen subjects and movements, highlighting the limited transferability of learned features in EMG-based models^26^. In contrast, subject-specific models align with individual neuromuscular signatures, enabling precise connections between kinematic inputs and muscle activations. While general models may offer scalability advantages, their effectiveness is limited and needs further support from the transfer learning domain. Overcoming these constraints requires adaptation strategies, such as domain-invariant feature learning, calibration-efficient transfer methods, or personalized fine-tuning on a small set of subject-specific data^26^. Furthermore, human motor control itself exhibits inter-subject variability, even when performing the same task, reflecting differences in neuromuscular coordination, joint synergies, and learned motor strategies. How to account for this variability is currently an active topic of discussion, as many studies still rely on averaging across participants. Recent studies highlight the importance of accommodating individual differences within synergetic motor control frameworks and caution against uncritical subject averaging, emphasizing that similar task outcomes do not guarantee identical internal control strategies^17,55,56^. Incorporating subject-specific analyses in future studies could therefore provide deeper insights into personalized motor coordination and improve model generalization across individuals.

The LSTM model applied in this work demonstrates a high degree of predictive accuracy for a range of movement types derived from the test dataset, see Fig. 4. Beyond achieving strong performance on known movement types, the model also exhibits the ability to generalize to new, previously unseen classes of motions. This generalization is crucial for several reasons. First, the ability to predict muscle activity for movements that were not explicitly included in training suggests that the model learns fundamental biomechanical principles rather than simply memorizing patterns of specific movement types. However, this ability to generalize comes at the cost of reduced accuracy compared to seen movements, reflecting the trade-off between model flexibility and specialization. This movement is particularly valuable for real-world applications such as rehabilitation, prosthetics, and human-machine interaction, where users frequently perform variations of known movements. Second, a model that generalizes well reduces the need for exhaustive datasets covering every possible movement range, allowing for efficient learning from limited training data while maintaining reliable performance on new tasks. Lastly, if the model can successfully predict muscle activation for new motions, it will further support the idea that neuromuscular patterns are governed by underlying kinematic constraints, contributing to a broader understanding of human motor control and movement strategies. In other words, the timing and amplitude of muscle activations are largely determined by the spatial and temporal requirements of the movement trajectory. While the prediction for the new motion dataset shows some limitations, the model successfully captures the timing of muscle activation well, though certain deviations appear in the corresponding amplitudes, see Fig. 8. This likely reflects that timing is more tightly linked to kinematic transitions, whereas amplitude depends more on subject-specific factors such as muscle strength, co-activation, and electrode placement variability, which were not explicitly modeled. These factors introduce nonlinear scaling effects that make amplitude prediction inherently more challenging. Notably, prediction accuracy is not consistent across all movement types. Certain new motions yield higher accuracy than others, and interestingly, movements that are typically considered simpler sometimes yield larger prediction errors than those that are considered more complex if removed from the training dataset. This trend is consistent across all subjects, despite utilizing subject-specific models. The model struggles more with new simple motions than with complex ones, even though both types of movement are nearly equally represented in numbers in the training dataset.

We further investigate the potential benefits of reducing redundancy in arm kinematics by incorporating the swivel angle to more accurately describe overall arm configuration. Without this additional parameter, the same EEF can correspond to multiple different arm configurations, potentially resulting in different neuromuscular activation patterns. In our study, subjects generally maintain a consistent elbow position within movement types, a finding that aligns with prior observations in undisturbed reaching tasks^46^. This consistency, however, does not necessarily hold for perturbed movements or specific grasping tasks, which are not part of our experimental design. Moreover, the initial arm posture is kept constant across trials. Prior research has demonstrated that the final configuration of the arm, particularly the orientation of the arm plane, is heavily influenced by the initial posture^57,58^. Additionally, studies on motor equivalence have shown that unpredictable perturbations during reaching movements lead to compensatory adjustments across multiple joints to maintain hand position, underscoring the flexibility and variability of elbow joint positioning^59^. From a conceptual standpoint, incorporating elbow-related information via the swivel angle offers a more complete representation of arm state. At the same time, our choice to model muscle activity based on EEF position and orientation is motivated by the functional relevance of the EEF to task execution. Many reaching studies have shown that the EEF is controlled rather than the joint configurations, as evidenced by the bell-shaped velocity profile of the EEF observed in reaching movements^60^. Although joint angles provide a unique and non-redundant representation of limb configuration, furthermore, more EEF-based representations have been shown to yield slightly better performance in predicting muscle activity^26^.

However, in the context of our specific experimental setup, the impact of including the swivel angle appears to be minimal. The paired *t*-test even reveals a significant reduction in performance when elbow information is added for the test and new motion dataset (Fig. 10). This outcome can be attributed to several factors. First, the variability in arm configuration within movement types is limited. Second, many of the movements in our dataset involve an extended arm or occur within a single plane, resulting in a naturally small swivel angle. Even when considering more complex movements where elbow dynamics play a role, the swivel angle are higher but still mostly consistent in one movement type. As a result, integrating this parameter into our framework yields negligible improvements in model performance. This suggests that our current experimental design may not be ideal for evaluating the relevance of additional elbow information. A more suitable setup would involve starting from a posture that is better defined by the swivel angle. For example, starting with the forearm flexed and the hand resting on a table, followed by reaching movements toward randomly placed objects, possibly under external perturbations. In such conditions, the contribution of the swivel angle would likely become more pronounced. Another approach involves specific movements where the arm is moved in such a way that the hand remains constant in space and orientation, but only the elbow is moved, which only activates the swivel angle and not the EEF.

We initially raised some fundamental issues that result from the redundancy inherent in the human motor control system, such as how multiple degrees of freedom (DOFs) are learned and coordinated. Is it more effective to first learn single-joint control and then combine these into multi-joint movements, or to acquire complex coordination directly? Related to this are the theories of movement primitives and submovements, which suggest that complex movements may be constructed from basic kinematic or dynamic units^32,33^. In early motor development, infants typically display multi-joint reaching behaviors^34^ rather than isolated joint control. During this stage, humans might rely on coarse motor synergies that are gradually refined with experience, reflecting a flexible integration of modular and global control strategies^17^. Initial reaching movements are often characterized by stiffening of the elbow and predominant use of shoulder musculature, possibly as a strategy to cope with arm redundancy. Over the first six months, the elbow gradually becomes more actively involved^61^, supporting a developmental progression from gross to fine motor skills^35^. Moreover, muscle activation for a given joint can differ depending on whether it is engaged in isolation or within a coordinated multi-joint task, emphasizing the importance of context-dependent neuromuscular control in movement execution. These considerations suggest that both modular decomposition and coordination principles are critical for understanding and modeling human motor control.

Our model performed significantly better when trained on the full dataset. However, training exclusively on complex multi-joint movements tends to result in better performance than training solely on simple single-joint movements. This could be attributed to the richer kinematic patterns present in complex movements, which allow the model to learn a more comprehensive representation of muscle activation dynamics. In contrast, simple movements may not provide sufficient variability for the model to generalize effectively. A notable consideration is that both the simple and complex models had only half the data for training compared to the model trained on both types. As mentioned in^26^, the impact of this reduced training data in a subject-specific model on the prediction ability is expected to be small but not negligible. The reduced number of data can explain why the models trained and tested exclusively on simple movements or complex ones, respectively, have slightly lower scores compared to the all model tested and the corresponding exclusive test datasets in Fig. 12.

Additionally, we explore the potential benefits of movement segmentation. The original hypothesis is that breaking down complex movements into kinematically simpler segments would facilitate learning by allowing the model to focus on smaller, more predictable patterns. However, our results show a significantly better performance without segmentation (Fig. 14), suggesting that either the segmentation approach based on inflection points is not optimal, or segmenting movement in general does not necessarily enhance model performance in this context. LSTMs are designed to learn temporal dependencies directly from sequential data, meaning that they can inherently detect motion transitions without the need for explicit segmentation, benefiting from long sets of data. If the model already effectively captures the underlying movement patterns, externally imposed segmentation does not provide additional benefits and could even disrupt the learned representations. One possible explanation is that the chosen segmentation method does not effectively capture meaningful transitions in movement. According to the literature, there are three primary types of boundaries to consider. Physical boundaries can be identified through changes in joint movement, as observed in gait analysis, such as the heel strike. Temporal boundaries consist of segments that are defined based on a template specified by the user. Lastly, derived metric boundaries can be established based on variations in the derived signal, similar to the approach we employed, or i.e., through the state transitions observed in Hidden Markov Models^62^. Or for instance, segmentation based on bell-shaped velocity profiles, as proposed in submovement theory^33^, where each submovement is considered an elementary unit. Such velocity-based segmentation may better reflect neuromuscular control strategies, as humans often plan movements in terms of velocity trajectories rather than absolute positions. Furthermore, in discrete movement tasks, as observed here, always starting and ending in a resting position, segmenting movements may offer little benefit if transitions between segments do not introduce significant variation in neuromuscular activation. In contrast, movement segmentation is likely to be more beneficial in continuous movement tasks, where muscle activation patterns evolve dynamically over time. Such scenarios are commonly encountered in real-world applications, including assistive technologies like exoskeletons and prosthetics, where understanding continuous movement phases is crucial for adaptive control. Future research should explore velocity-based segmentation and strategies tailored to these applications, potentially integrating biomechanical insights to refine segment boundaries and improve learning outcomes.

A critical consideration in interpreting our results is the balance between model-related limitations and the inherent variability of human motor control. From the modeling perspective, the LSTM primarily learns statistical regularities from the training data, which allows robust performance when predicting movements within familiar contexts but limits its ability to extrapolate to unseen movements. From a motor control perspective, variability in task context, coordination strategies, and muscle recruitment adds additional complexity. Even biomechanically similar movements can differ subtly in timing, joint synergies, or neural strategies, which may prevent straightforward generalization from one task to another. Nevertheless, the degree of variability in the present dataset was reduced by the relatively repetitive and controlled nature of the experimental tasks, as well as by the reliance on EMG signal as the primary feature, which provides only a partial representation of neuromuscular control.

For future research, it would be valuable to investigate whether the proposed model can be extended to physical interaction tasks, such as holding or transporting objects. In this context, the influence of gravity and task dynamics is particularly relevant, as variations in movement direction are known to systematically affect hand kinematics and muscle activation^63^. This will likely require additional inputs, such as end effector forces or torques from a musculoskeletal model, and a dataset combining free-reaching and interaction tasks. Such work would address fundamental questions on how EMG maps to both motion and force, and could advance neuromuscular modeling for applications in rehabilitation, assistive robotics, and exoskeleton control.

## Conclusions

This study explores the relationship between upper limb kinematics and muscle activity using an LSTM model trained on end effector (EEF) data. The model demonstrates strong predictive accuracy for known movements and generalizes to unseen movements, highlighting its ability to learn fundamental biomechanical principles rather than memorizing specific patterns. This generalization is valuable for applications in rehabilitation and human-machine interaction, reducing the need for exhaustive datasets. However, prediction accuracy varies across movements, with some unseen simple movements being harder to predict than complex ones, suggesting that execution variability or kinematic distinctiveness may play a role.

We also examine movement segmentation, hypothesizing that breaking movements into simpler parts could enhance learning. Our results, however, indicate that the LSTM was able to capture movement transitions without requiring additional segmentation, as the inflection-point–based approach did not yield measurable performance improvements.

Finally, alongside the EEF, we investigate the role of the swivel angle in reducing redundancy in arm kinematics. Although including elbow information provides a more complete representation of the arm’s configuration, it has a small impact on reaching movements in our study. This is because such movements are typically performed with a consistent arm configuration, with only minimal variation in the swing angle. This observation aligns with previous research showing that arm configuration remains stable during undisturbed reaching tasks.

## Supplementary Information

Supplementary Information.

## Data Availability

The datasets used and/or analysed during the current study are available from the corresponding author on reasonable request.
