# Supplementary material for: Insights into motor control: predict muscle activity from upper limb kinematics with LSTM networks
Source: Sci Rep. 2026 Jan 5;16:2614. doi: 10.1038/s41598-025-33696-y (PMC12820307; doi:10.1038/s41598-025-33696-y)
Supplement: Supplementary file 1 — Supplementary Information. [file 41598_2025_33696_MOESM1_ESM.pdf]

Supplementary Information

Supplementary Table 1: Overview of the LSTM model trained on all data types (all) performance for the new motion dataset under various conditions: entire movement sequences (1seq), without elbow information (0elbow), and with elbow information (1elbow). The performance is evaluated by the zero-line score Z_s, mean squared error MSE, the squared correlation coefficient r^2, and the coefficient of determination R^2. Movements considered with crucial elbow information are wave, breaststroke, and reading a watch. Movements with no crucial elbow information are shoulder abduction, shoulder flexion, wrist pronation, and elbow extension flexion.

	dataset	new complex		new simple	
		$1seq, 1elbow$	$1seq, 0elbow$	$1seq, 1elbow$	$1seq, 0elbow$
new prediction	Z_s	45.77	45.90	51.45	**56.57**
	MSE	0.0083	0.0076	0.0034	0.0032
	r^2	0.54	0.57	0.59	0.65
	R^2	0.0874	0.1275	0.0772	0.2440

Supplementary Table 2: Overview of the LSTM model performance trained on entire movement sequences (1seq), without elbow information (0elbow), evaluated using the zero-line score Z_s, mean squared error MSE, the squared correlation coefficient r^2, and the coefficient of determination R^2 across all data types (all), simple data types (simple), and complex data types (complex).

	dataset	simple		complex	
	trained on	all	simple	all	complex
test prediction	Z_s	83.61	77.14	**85.81**	85.21
	MSE	0.0019	0.0025	0.0029	0.0033
	r^2	0.81	0.74	0.77	0.76
	R^2	0.7948	0.70239	0.7564	0.7507
new prediction	Z_s	**45.09**	28.81	42.43	37.16
	MSE	0.0051	0.00539	0.0061	0.0086
	r^2	0.61	0.57	0.55	0.39
	R^2	0.0742	-0.2374	0.1277	-0.0928

Supplementary Table 3: Performance of the LSTM model trained on entire movement sequences (1seq), and sub-sequences (nseq), without elbow information (0elbow), and with elbow information (1elbow), evaluated using the Z_s score, mean squared error MSE, the squared correlation coefficient r^2, and the coefficient of determination R^2 across all data types (all).

	score	*nseq0elbow*	*nseq1elbow*	*1seq0elbow*	*1seq1elbow*
test prediction	Z_s	82.25	79.42	**84.27**	82.52
	MSE	0.0026	0.003	0.0025	0.0030
	r^2	0.78	0.74	0.79	0.76
	R^2	0.76	0.72	0.78	0.74
new prediction	Z_s	43.51	39.77	**47.63**	45.28
	MSE	0.0045	0.0049	0.0045	0.0048
	r^2	0.49	0.47	0.52	0.52
	R^2	0.0066	-0.0371	0.0886	-0.0359

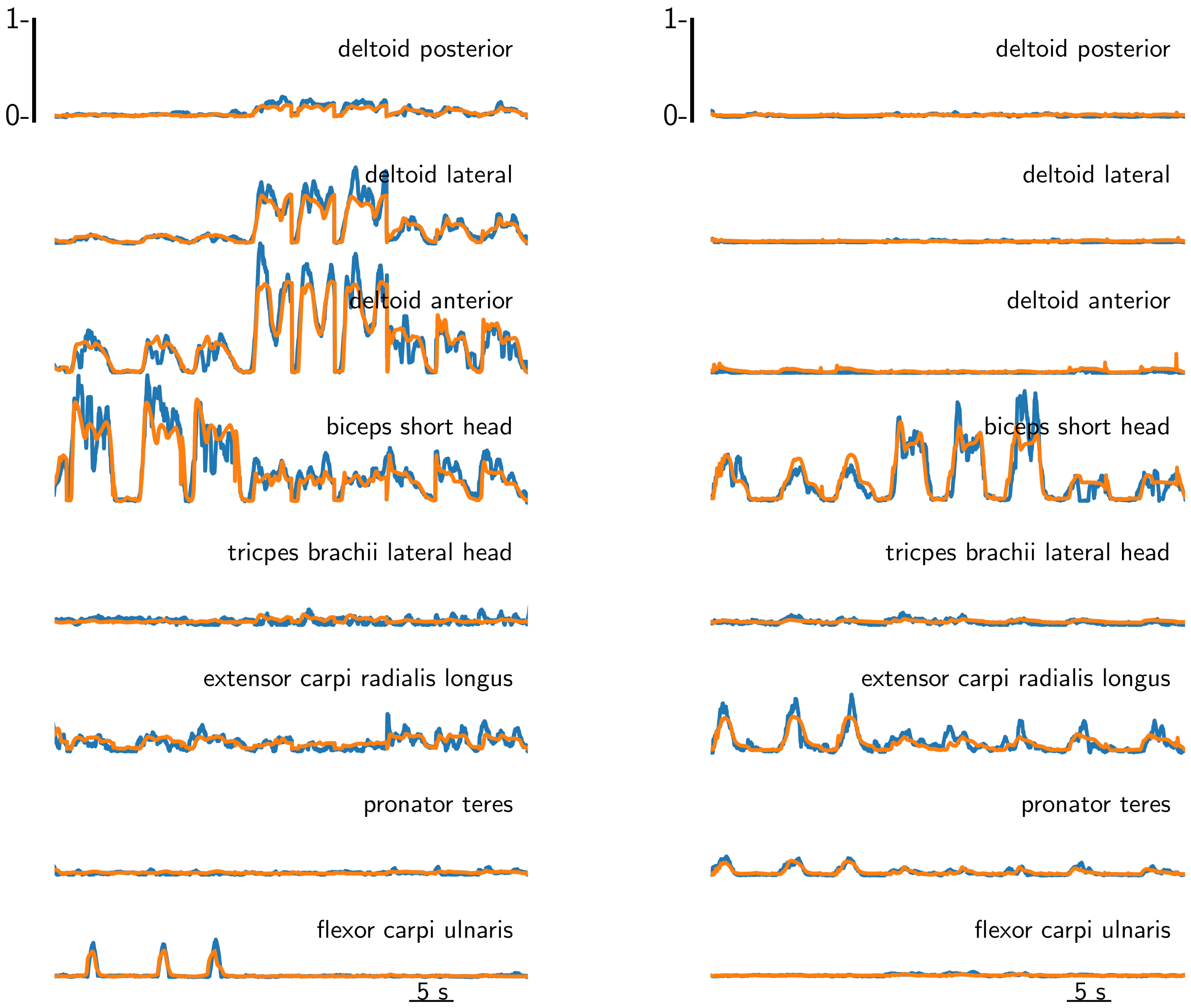

Supplementary Figure 1: Performance of the LSTM model trained on entire movement sequences (1seq), without elbow information (0elbow). An example prediction from the test dataset with left) the breaststroke ($Z_s = 85.53$, MSE $= 0.0025$, $r^2 = 0.79$, $R^2 = 0.77$) and right) reading a clock ($Z_s = 88.38$, MSE $= 0.0022$, $r^2 = 0.83$, $R^2 = 0.82$) illustrates the recorded muscle activity for all eight EMG channels (blue), alongside the model generated muscle activity (orange).

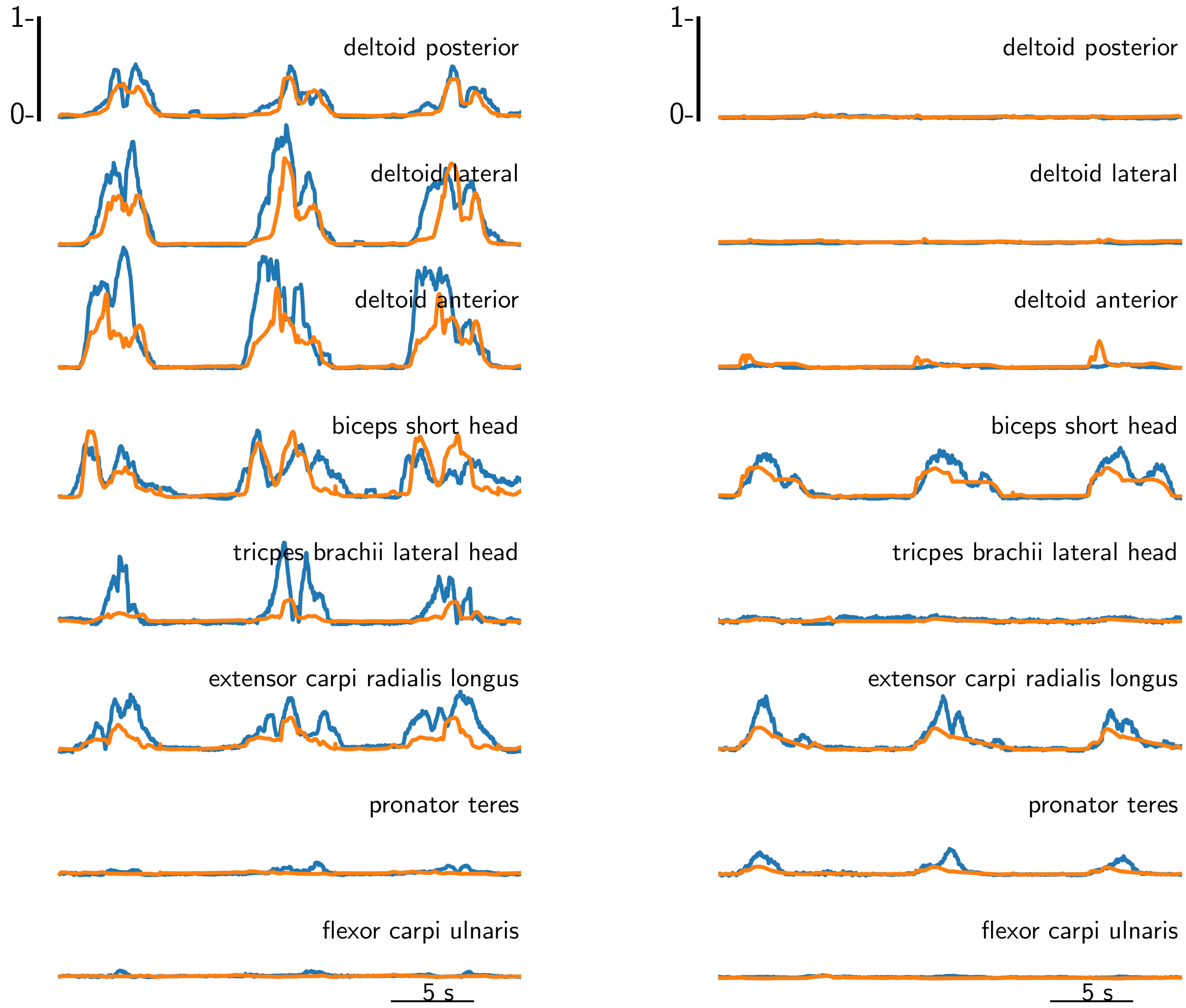

Supplementary Figure 2: Performance of the LSTM model trained on entire movement sequences (1seq), without elbow information (0elbow). An example prediction from the new motion dataset with left) the breaststroke ($Z_s = 68.22$, MSE $= 0.0097$, $r^2 = 0.61$, $R^2 = 0.42$), and right) elbow flexion ($Z_s = 71.12$, MSE $= 0.001$, $r^2 = 0.68$, $R^2 = 0.52$) illustrates the recorded muscle activity for all eight EMG channels (blue), alongside the model generated muscle activity (orange).

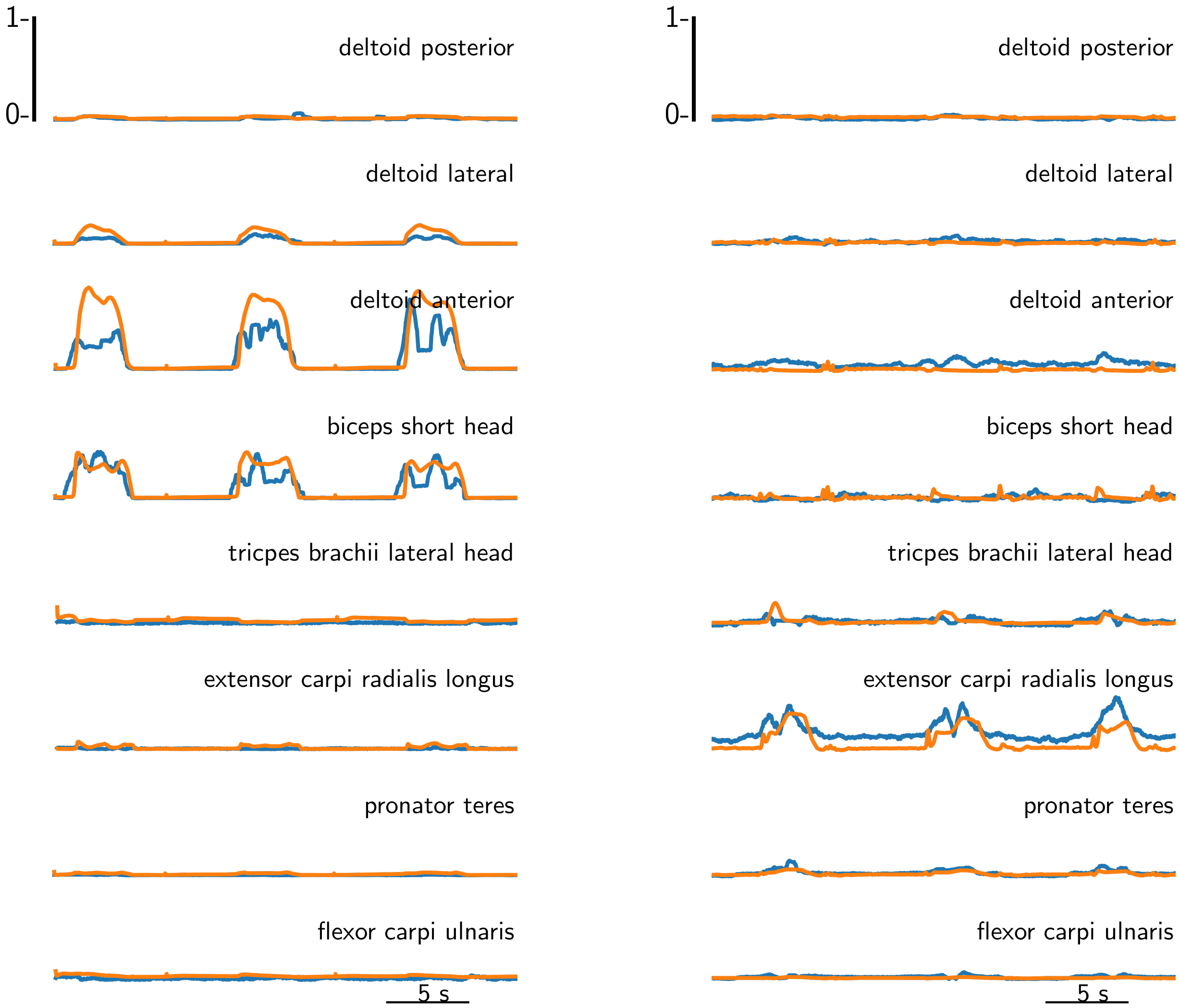

Supplementary Figure 3: Performance of the LSTM model trained on entire movement sequences (1seq), without elbow information (0elbow). An example prediction from the new motion dataset with left) the shoulder flexion (mix) ($Z_s = 24.98$, MSE $= 0.0025$, $r^2 = 0.69$, $R^2 = -0.18$), and right) wrist pronation ($Z_s = 61.23$, MSE $= 0.001$, $r^2 = 0.29$, $R^2 = -0.85$) illustrates the recorded muscle activity for all eight EMG channels (blue), alongside the model generated muscle activity (orange).

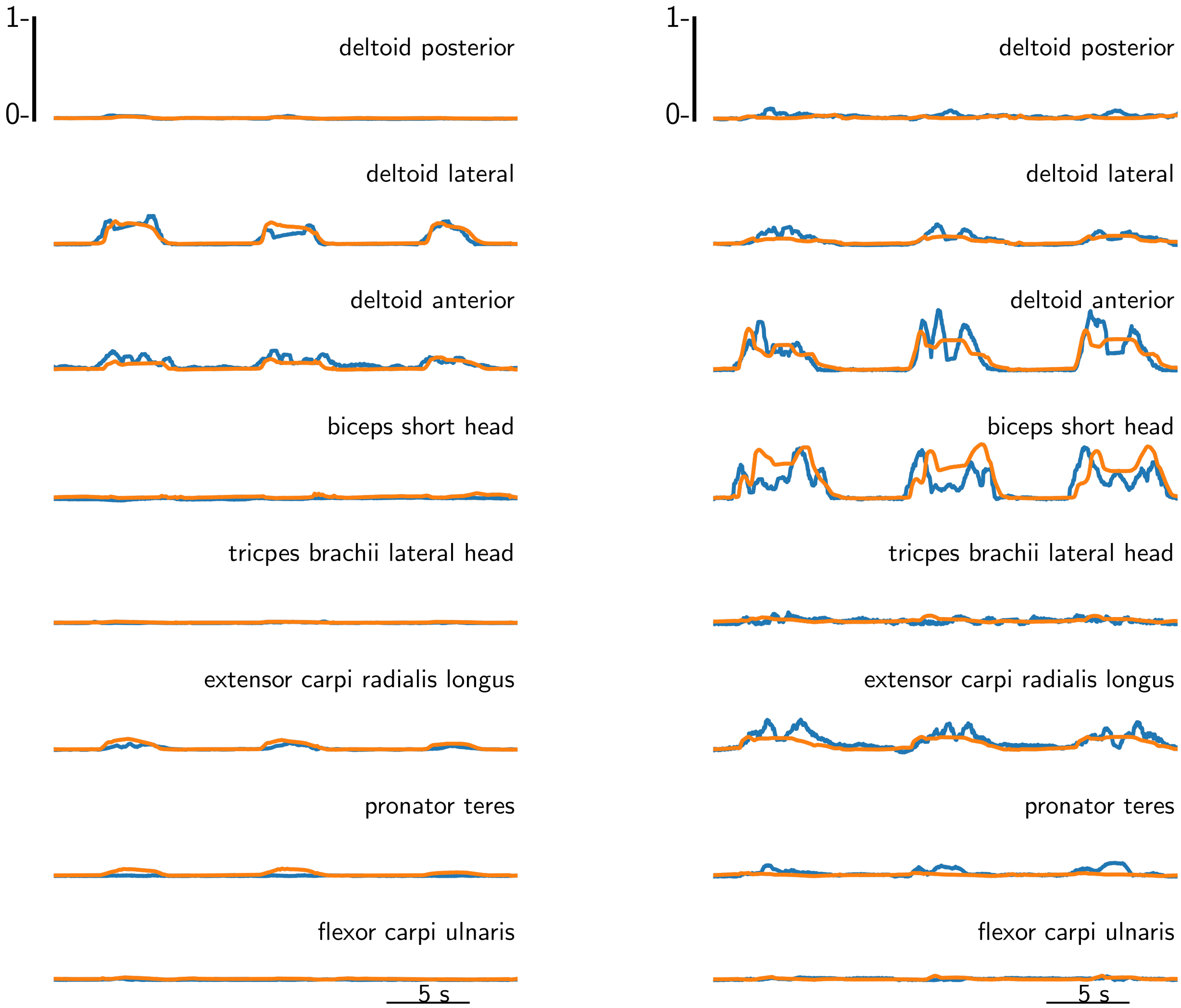

Supplementary Figure 4: Performance of the LSTM model trained on entire movement sequences (1seq), without elbow information (0elbow). An example prediction from the new motion dataset with left) the shoulder abduction mix ($Z_s = 71.34$, MSE $= 0.0002$, $r^2 = 0.68$, $R^2 = 0.52$,) and right) reading a clock ($Z_s = 58.43$, MSE $= 0.0024$, $r^2 = 0.51$, $R^2 = 0.21$) illustrates the recorded muscle activity for all eight EMG channels (blue), alongside the model generated muscle activity (orange).

Supplementary Table 4: Results of the Shapiro-Wilk test assessing normality of the z-transformed data across figures. The critical value for $n = 5$ and $p = 0.05$ is set to $W_{\text{critical}} = 0.762$, and values below this threshold indicate a significant deviation from the normal distribution.

**Figure**	**Statistic W**	**p-value**
Fig. 10	0.9878	0.9716
	0.9598	0.8069
Fig. 11	0.8768	0.2952
	0.9174	0.5133
Fig. 12	0.9888	0.9752
	0.9422	0.6814
	0.9807	0.9382
	0.9028	0.4257
	0.9841	0.9554
	0.9812	0.9408
Fig. 14	0.9878	0.9716
	**0.7479**	**0.0284**
	0.8417	0.1697
	0.9872	0.9692
	0.9598	0.8069
	0.8902	0.3582
	0.8883	0.3485
	0.8842	0.3289
